# A soft 3-DOF interaction force measurement system for estimating the biomechanical effects of a soft wearable robot on the human joint

**DOI:** 10.1017/wtc.2025.10014

**Published:** 2025-07-15

**Authors:** Seongyun Cho, Byungjun Jeon, Minki Kim, Seongok Chae, Seungmin Ye, Yoo-Jin Jun, Yong-Lae Park, Hyung-Soon Park

**Affiliations:** 1 Department of Mechanical Engineering, Korea Advanced Institute of Science and Technology, Daejeon, Republic of Korea; 2 Department of Mechanical Engineering, Seoul National University, Seoul, Republic of Korea

**Keywords:** wearable robot, exosuit, biomechanics, force sensors, interaction force, physical human-robot interaction

## Abstract

Recent advancements in wearable robots have focused on developing soft, compliant, and lightweight structures to provide comfort for the users and to achieve the primary function of assisting body motions. The interaction forces induced by physical human-robot interaction (pHRI) not only cause skin discomfort or pain due to relatively high localized pressures but also degrade the wearability and the safety of the wearer’s joints by unnaturally altering the joint reaction forces (JRFs) and the joint reaction moments (JRMs). Although the correlation between excessive JRFs/JRMs and joint-related conditions has been reported by researchers, the biomechanical effects of forces and moments caused by the pHRI of a wearable robot on the wearer’s joints remain under-analyzed. In this study, we propose a method of measuring and analyzing these interactions and effects, using a custom-designed soft, three-degree-of-freedom (DOF) force sensor. The sensor is made of four Hall effect sensors and a neodymium magnet embedded in a silicone elastomer structure, enabling simultaneous measurement of normal and two-axis shear forces by detecting the distance changes between the magnet and each Hall effect sensor. These sensors are embedded in contact pads of a commercial wearable robot and measure the interaction forces, used for calculating JRF and JRM. We also propose a modified inverse dynamics approach that allows us to consider the physical interactions between the robot and the human body. The proposed method of sensing and analysis provides the potential to enhance the design of future wearable robots, ensuring long-term safety.

## 1. Introduction

The wearability of wearable robots is becoming a critical factor in design due to the recent hardware transitions from rigid exoskeletons to soft exosuits (Bartenbach et al., 2015; Liu et al., 2021; Cao et al., 2021). This shift aims to enhance user comfort and acceptance by utilizing flexible, lightweight materials, such as cable-driven actuators or fluidic artificial muscles to assist human movements (Thalman and Artemiadis, 2020; Gonzalez-Vazquez et al., 2023). Assistance forces and moments provided by wearable robots are transferred to the human body as interaction forces through soft pads, braces, or cushions (Asbeck et al., 2015; Wilcox et al., 2016; Lee and In, 2023). However, misalignment or overtightening can yield misdirected or excessive interaction forces (d’Elia et al., 2017; Langlois et al., 2020), which not only hinder effective assistance but also have a negative impact on the wearability due to discomfort or pain at the contact interfaces (d’Elia et al., 2017; Serrancolí et al., 2019; Thalman and Artemiadis, 2020; Wang et al., 2022). In this regard, measuring interaction forces from physical human-robot interaction (pHRI) is important not only to ensure performance but also to monitor the wearability for long-term use of wearable robots (Tepe et al., 2017).

Although previous studies have addressed the interaction forces caused by pHRI (Lenzi et al., 2011; Rathore et al., 2016; Long et al., 2017; Georgarakis et al., 2018; Bessler et al., 2019; Leal-Junior et al., 2019; Langlois et al., 2020), only a few of them explored the biomechanical effects on human joints. To date, research on pHRI primarily focused on improving comfort (Lenzi et al., 2011; Rathore et al., 2016) or control performance (Ka et al., 2016). However, interaction forces at the contact area also affect the joint reaction forces (JRFs) and moments (JRMs) (Kingma et al., 1996; Andersen and Rasmussen, 2023; Van der Kooij et al., 2022), which should be taken into account in design, since increased forces or moments on human joints beyond typical daily levels are closely associated with an increased risk of injuries on joints (Kumar, 2001; Amin et al., 2004; Wearing et al., 2006; Lewis et al., 2007; Sun, 2010; D’Lima et al., 2012). Monitoring the wearer’s JRFs and JRMs is therefore essential for evaluating the long-term safety of wearable robots. To support this, continuous measurement of interaction forces in full degrees of freedom (DOF) on the wearer’s body is essential in assessing their biomechanical effects on the human joints.

For accurate estimation of JRFs and JRMs in human joints, the conventional inverse dynamics approach (Kingma et al., 1996; Chowdhury and Kumar, 2013; Baltzopoulos, 2024; Caruntu and Moreno, 2019; Derrick et al., 2020) should be modified to include 3-DOF interaction forces between the wearable robot and the wearer. The conventional method did not account for the interaction forces, the wearer and the robot were regarded as a single rigid body without relative movements. Consequently, the equations of motion are derived for combined segments (Ka et al., 2016; Bergmann et al., 2022), resulting in JRFs and JRMs shared by the joints of the human and the robot simultaneously, rather than considering them as the forces acting solely on the human joints. To decouple the human body from the wearable robot, interaction forces should be measured and treated as external forces acting on the individual human body segments in the equations of motion. Therefore, we propose a modified inverse dynamics approach that incorporates the measured 3-DOF interaction forces. The computed JRFs and JRMs from the modified inverse dynamics reflect the actual forces and moments applied exclusively to the wearer’s joints, yielding more accurate results than those from the conventional methods.

Although various force or pressure sensors have been used to estimate interaction forces between the human body and the wearable robot (Tomo et al., 2016; Paulino et al., 2017; Billeschou et al., 2021), embedded sensors at the contact area were mostly limited to measuring single-axis normal forces without shear components (Mirzanejad and Agheli, 2019). As a result, they are insufficient to provide accurate JRFs and JRMs necessary for precise biomechanical analysis. While conventional multi-axis force/torque sensors (Kim et al., 2020; Cao et al., 2021) are available, they are unsuitable for embedding in contact areas due to their rigidity, bulky size, and weight. Soft multi-axis piezoresistive force sensors using liquid metal and silicone elastomer have also been developed (Vogt et al., 2013; Kim and Park, 2018), but they are not compact enough to be easily embedded in wearable robots and also show nonlinear responses due to hysteresis. In contrast, Hall effect-based soft sensors capable of measuring 3-DOF forces show high linearity with low hysteresis and provide compact form factors (Natale and Torres-Jara, 2006; Nie and Sup, 2017; Dwivedi et al., 2018). Also, commercial Hall effect sensors are widely available and economical, enabling flexible design choices in terms of performance and cost (Nasab et al., 2022). We thus employed Hall effect sensors combined with a magnet in a soft structure to meet the following requirements: 3-DOF (normal and shear) force measurement, structural flexibility, lightweight, compact form factor, and an untethered system. In this study, each 3-DOF sensor unit, comprising four Hall effect sensors, was fabricated in approximately 1.5 hr, with a footprint of 15 × 20 mm and a weight of 4 g. Given the large number of units needed to cover all contact areas, using a highly cost-effective option was a practical choice.

**Figure 1.**
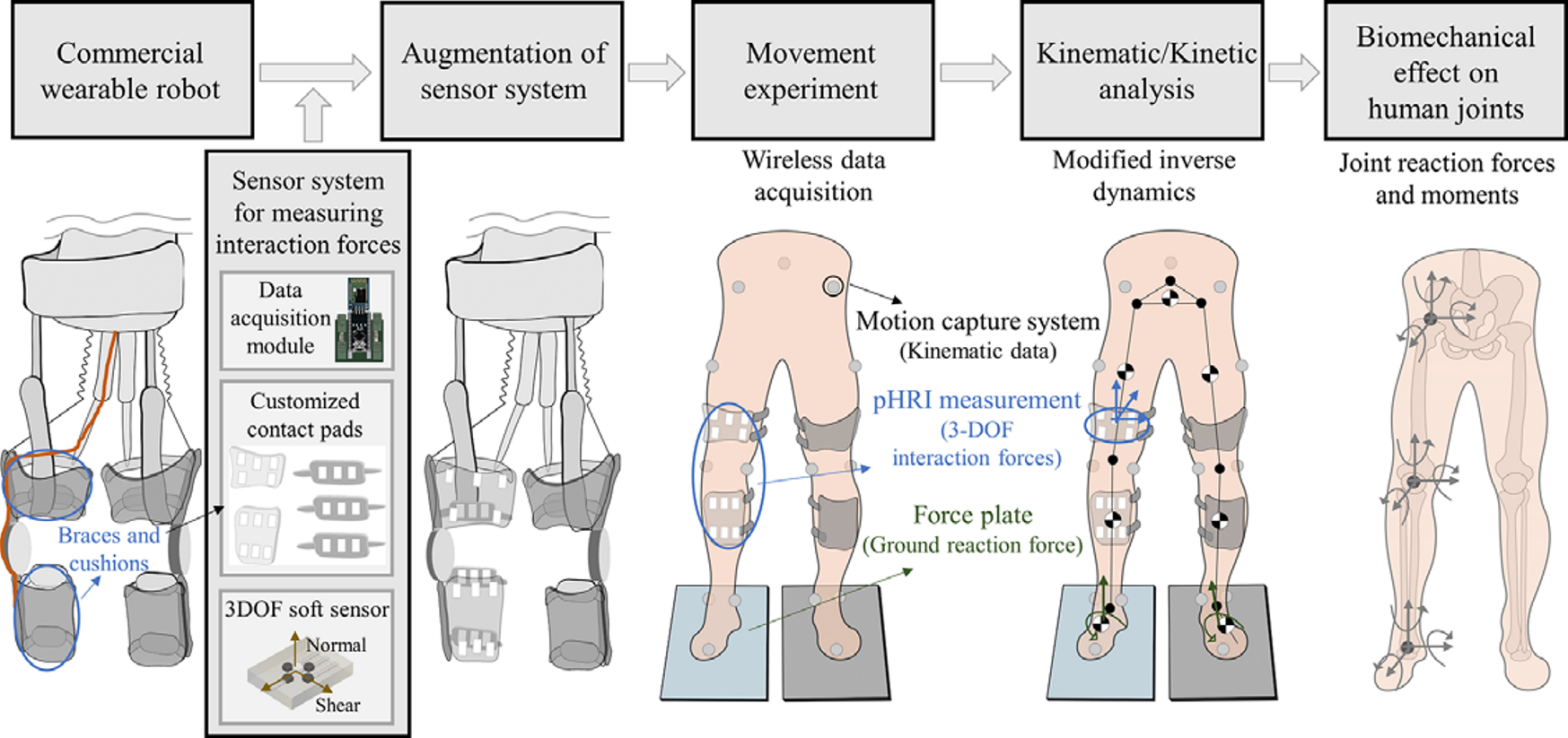
System methodology overview. The sensor system for measuring full DOF interaction forces was augmented to the commercial wearable robot by replacing original physical interfaces with customized contact pads. A movement experiment was subsequently conducted utilizing the Motion capture system, force plate, and pHRI measurement system. The modified inverse dynamics incorporates these interaction forces from the pHRI into the calculation of joint reaction forces and moments, enabling an accurate assessment of the biomechanical effects of the wearable robot on human joints.

In this paper, interaction forces due to pHRI at contact areas were measured by our soft 3-DOF force sensor system, with which the biomechanical effect of robotic assistance on human joints could be estimated, as shown in Figure 1. The braces and soft pads of a commercial wearable robot were replaced with custom-built contact pads embedded with a pHRI measurement system, composed of 3-DOF soft sensors and data acquisition modules. The feasibility of our system was experimentally validated on five healthy subjects with a commercial exosuit. Using modified inverse dynamics, we observed significant differences in JRFs and JRMs in certain directions that could not be identified by conventional inverse dynamics analysis. Therefore, our study (1) provides a practical method for continuous measurement of 3-DOF interaction forces using custom-designed contact pads, applicable to various wearable robots without structural or functional modifications; and (2) presents the modified inverse dynamics method that accurately estimates JRFs and JRMs of the wearer’s joints, enabling precise biomechanical analysis essential for long-term safety assessment.

## 2. Materials and methods

### 2.1. Soft three-axis force sensor system

#### 2.1.1. Design and fabrication

**Figure 2.**
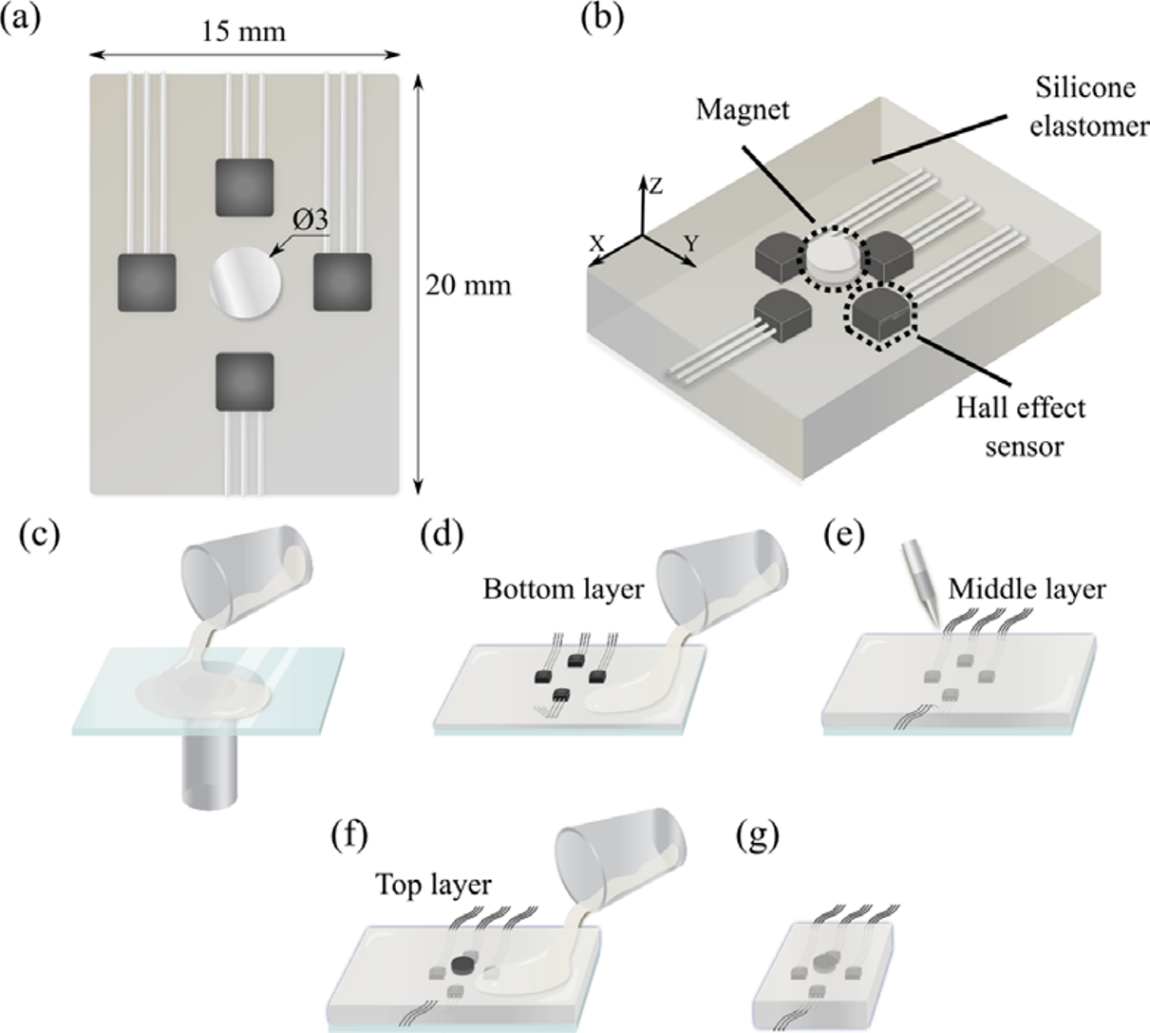
Sensor design and fabrication procedure. (a) Dimension from the top view and (b) components of the sensor. (c) Silicone elastomer is poured on a glass plate and cured. (d) Four Hall effect sensors are fixed in position and the silicone elastomer is poured on top and cured. (e) Wires are soldered at Hall effect sensors. (f) Magnet is placed at the center and silicone elastomer is poured on top and cured. (g) Sensor is cut into an appropriate size.

The proposed sensor measures normal force along the z-axis and shear forces acting in the x–y plane (Figure 2). The distance between each Hall effect sensor and the permanent magnet changes depending on the applied force. When a normal force is applied, the distance between each Hall effect sensor and the magnet is decreased, leading to an increased sensor output. When a shear force is applied in either the x- or y-direction, the sensors aligned perpendicular to the force move farther from the magnet, increasing their distance from it. Meanwhile, the two sensors aligned along the force direction become either closer or farther from the magnet, depending on the direction of the applied force.

The sensing capability of both normal and shear forces is achieved by the sensor structure, composed of three layers of silicone elastomer: a permanent magnet on top, only elastomer material in the middle to allow deformation, and four Hall effect sensors at the bottom (Figure 2(b)). The dimensions of the sensor are 15 mm (width), 20 mm (length), and 5 mm (thickness), as shown in Figure 2(a). The top layer consists of a magnet and silicone elastomer. A high-strength neodymium magnet was used to minimize unwanted influences from external magnetic fields. The magnetic field created by the embedded magnet can be detected by the Hall effect sensors embedded in the bottom layer. This layer needs to be sufficiently deformable to ensure sensitivity to forces in multiple directions. Therefore, the top layer that encloses the magnet was made from a more compliant elastomer material than that of the bottom layer. The middle layer with no embedded components is used for pure deformation. Since the deformation of this layer is directly related to the sensor sensitivity, a relatively soft silicone elastomer was used. The bottom layer consists of four Hall effect sensors embedded in silicone elastomer. These Hall effect sensors should remain in the same position during application of external forces, since their movement can cause inaccurate force estimation. A relatively stiff silicone elastomer was thus used to minimize the unwanted movements of the sensors embedded in the bottom layer.

The soft force sensor was fabricated by sequentially assembling three different layers. Starting from the bottom layer with four Hall effect sensors, the middle layer for deformation, and the top layer with a permanent magnet, the layers were stacked in sequence. The fabrication process is shown in Figures 2(c–g). First, a thin layer of silicone elastomer (Dragon Skin 30, tensile strength: 6.55 MPa, Shore hardness: 30A, Smooth-On Inc.) is spin-coated on a glass plate and cured in an oven for 30 min (Figure 2(c)). A blueprint for alignment is then attached to the bottom of the plate and four Hall effect sensors (WSH-135, Winson Semiconductor Corp.) are fixed in place using a silicone adhesive (Sil-Poxy, Smooth-On Inc.) above the blueprint (Figure 2(d)). Another layer of silicone elastomer is spin-coated directly and oven-cured for 30 min, completing the bottom layer. The Hall effect sensors are soldered with electrical wires, and silicone elastomer (Dragon Skin 10, tensile strength: 3.79 MPa, Shore hardness: 10A, Smooth-On Inc.) is spin-coated and cured to form the middle layer (Figure 2(e)). A neodymium magnet is then placed at the center, and a silicone elastomer layer is spin-coated on top and cured in an oven, forming the top layer (Figure 2(f)). Finally, the sensor is cut to an appropriate size (Figure 2(g)).

#### 2.1.2. Force characterization

**Figure 3.**
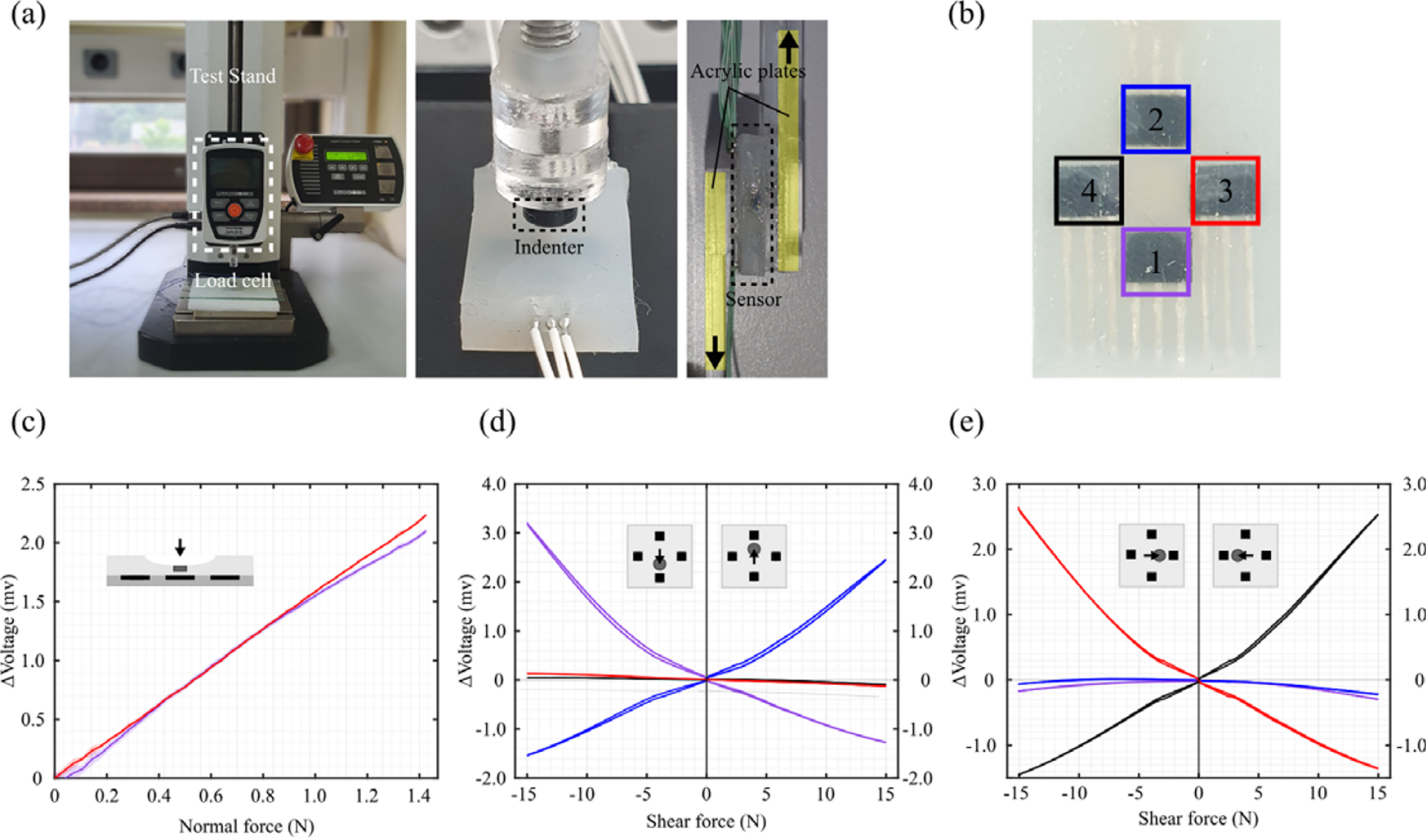
Sensor experimental setup and results for characterization. (a) Experimental setup for normal and shear force testing. A 5 mm diameter indenter was used to apply the normal force, while acrylic plates were attached to the sensor surface for the shear force tests. (b) Hall sensor layout showing sensors 1 through 4, each marked with a different colored box. (c) Normal force characterization results for sensor 1 and 3. (d) Shear force characterization results in the directions corresponding to sensors 1 and 2. (e) Shear force characterization results in the directions corresponding to sensors 3 and 4.

Sensor characterization was conducted to obtain the sensor response under repetitive normal and shear forces in different directions. The maximum values of 200 and 150 kPa for normal and shear stresses, respectively, were selected, based on the preliminary experiments on the range of interaction pressures using the wearable robot. The maximum stresses were large enough to exceed the measured maximum value in the wearable robot as well as the safety thresholds for pain and blister prevention discussed in previous studies (Naylor, 1955; Mao et al., 2017; Kermavnar et al., 2018; Bessler et al., 2021). For the normal force test, a cylindrical indenter with a diameter of 3 mm was attached to the end of the load cell (Series 3, Mark-10) installed in a tensile tester (ESM 303, Mark-10), and the sensor was placed under the indenter (Figure 3(a)). Each Hall effect sensor was marked from 1 to 4, as indicated in Figure 3(b). The experiment was conducted by pressing and releasing the top surface of the sensor using the indenter ten times. Due to the symmetric configuration of the Hall effect sensors, only the data from sensors 1 and 3 were collected. During the experiment, the maximum applied normal force was approximately 1.4 N, corresponding to a normal stress of 200 kPa. It can be seen from Figure 3(c) that the output of each Hall effect sensor increased as the normal force increased.

For the shear force test, two acrylic plates were bonded on the top and the bottom surfaces of the sensor to measure the output signals with shear forces (Figure 3(a)). The bottom plate was firmly fixed to the test stand. The top plate was moved in-plane to create shear forces in different directions. The experiment was conducted moving the top plate upward and downward ten times each, with the maximum shear force of approximately 15 N, corresponding to a shear stress of 150 kPa. It can be seen from Figure 3(d) and (e) that the sensor outputs of the Hall effect sensors placed in the force direction either increased or decreased with opposite trends. This is because the magnet moves away from one Hall effect sensor while it moves closer toward the other Hall effect sensor on the same axis. A cyclic test was also conducted 1,000 times for both the normal and the shear forces to assess repeatability and stability (Supplementary Figure S1).

#### 2.1.3. Integration with a wearable robot

Our proposed sensor system was integrated into a commercial wearable robot, (Myosuit, MyoSwiss AG), to measure 3-DOF interaction forces at lower limb contact areas (Haufe et al., 2019; Koginov et al., 2022). Customized contact pads, closely modeled after the braces and cushions of Myosuit’s contact interfaces (Figure 5(a)), were fabricated using the same silicone material (Dragon Skin 30) as the 3-DOF sensors to ensure consistent mechanical properties. The sensor numbers and locations were determined by the contact areas and protruded regions of the interfaces. Each contact pad contained three evenly spaced sensors, with the central sensor positioned at the most anterior or posterior point of the body segment. Only the anterior thigh pad, with a larger contact area and more protrusions, had five sensors. The number of sensors can be adjusted depending on the pad designs. The sensor thickness slightly exceeded the depth of the pad cavities to facilitate interaction force transfer. These customized contact pads were integrated into the right limb of the wearable robot (Figure 5(c)). This augmentation increased the overall weight by 0.49 kg, less than 10% of the original weight of the robot. To evaluate potential changes in the wearer’s movements caused by embedding the sensor system, joint kinematics were analyzed during sit-to-stand, overground walking, and stair-climbing tasks (Supplementary Table S1 and Supplementary Figure S2). The differences were observed in only a few lower limb joint angles (4 out of 54) during these tasks.

#### 2.1.4. Data acquisition module

**Figure 4.**
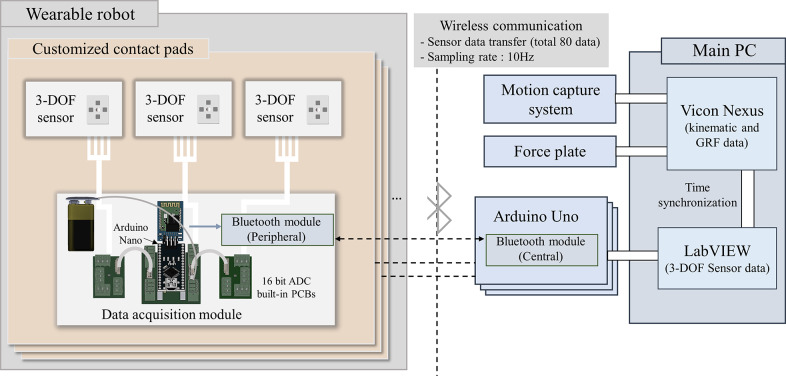
Schematic diagram for wireless sensor data acquisition. Each contact pad integrates the sensor system consisting of a data acquisition module and 3-DOF soft sensors. The data acquisition module reads the sensor output via a direct connection between each Hall effect sensor of the 3-DOF sensor and a 16-bit ADC. Each data acquisition module is wirelessly connected to an Arduino Uno via Bluetooth for sensor data transfer. A total of 80 sensor outputs from all contact pads were transmitted at a sampling rate of 10 Hz and synchronized with the Motion capture system.

**Figure 5.**
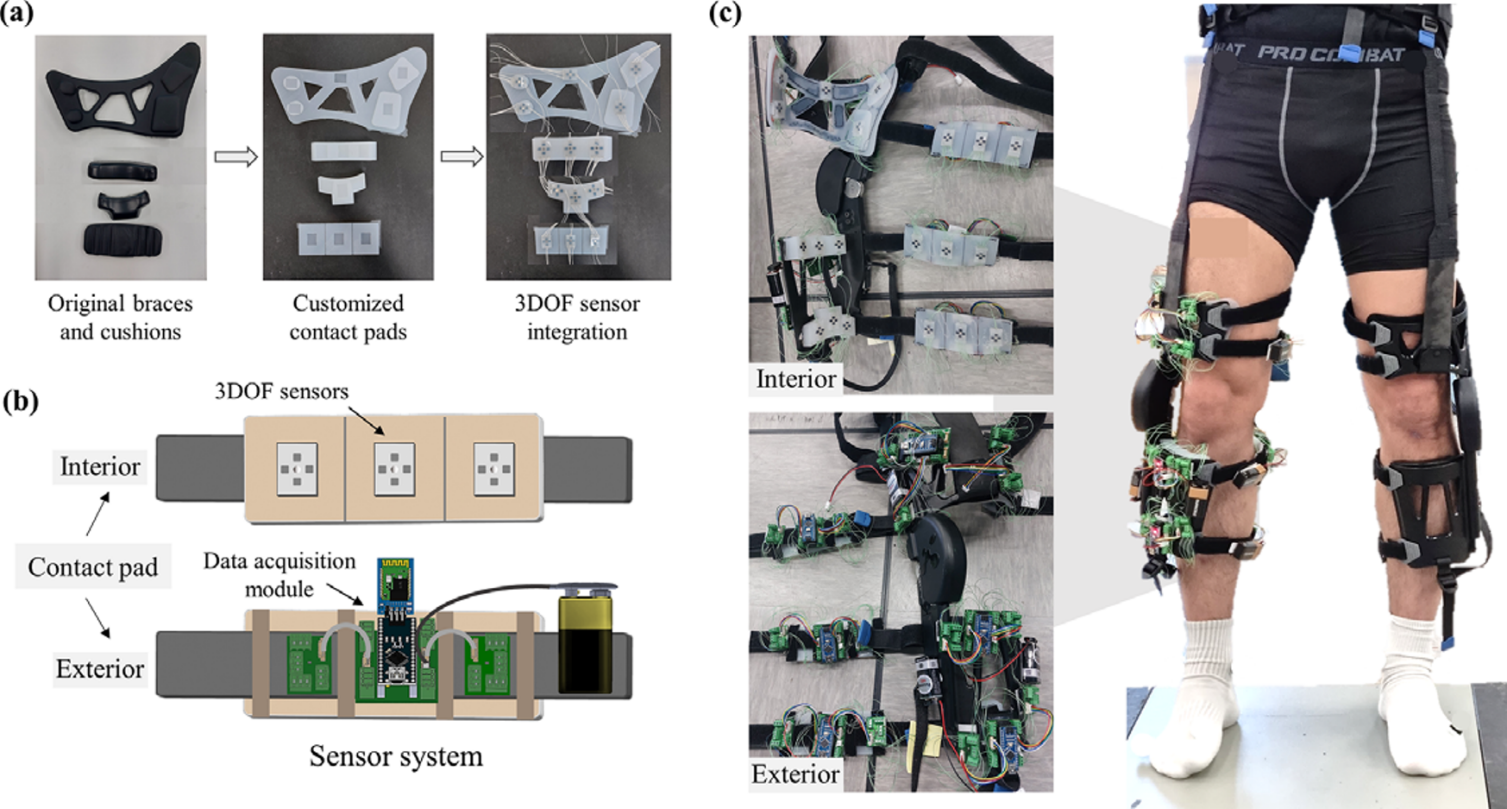
Augmentation of the sensor system on a commercial wearable robot. (a) The customized soft contact pads replicate the original braces and cushions while providing additional spaces for the fixation of 3-DOF force sensors. (b) Illustrations of the customized contact pad integrated with the sensor system, showing the 3-DOF sensors and data acquisition module positioned on the interior (contact areas) and exterior, respectively. (c) Images depicting the interior and exterior views of the right limb of the commercial exosuit (Myosuit) after attachment of the customized contact pads (Left). A photograph illustrating the subject wearing the exosuit with the sensor system is presented (right). Six contact pads, each equipped with a total of twenty 3-DOF sensors, cover all contact regions on the right lower limb.

To enable wireless data transmission via Bluetooth, we implemented a data acquisition module that transmits the Hall effect sensor signals to the main computer. Each module consists of a four-channel 16-bit analog-to-digital converter (ADC) (ADS1118, Texas Instruments) for reading the outputs from the four Hall effect sensors embedded in a single 3-DOF force sensor, along with an Arduino Nano, a Bluetooth module (HC-06, JN Huamao Technology Co., Ltd.), and a 9 V battery for data transmission and power supply (Figure 4). Each custom-built contact pad is equipped with its own data acquisition module, with each ADC assigned to a single 3-DOF sensor. The number of ADCs corresponds to the number of sensors in each pad. All the contact pads, except for the anterior thigh one with five ADCs, contain three ADCs for three embedded sensors. These modules are externally mounted on the contact pads (Figure 5(b)) to avoid movement interference.

### 2.2. Experimental protocol

**Figure 6.**
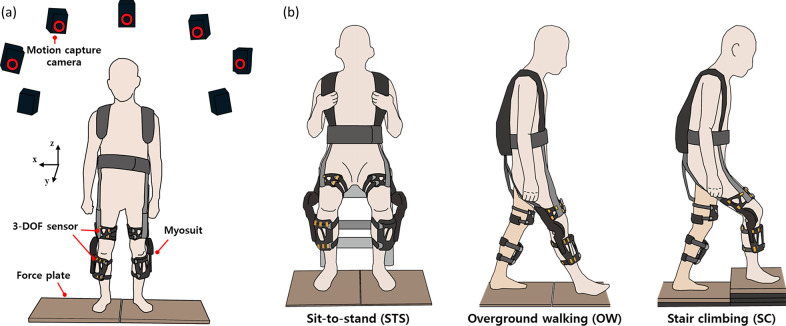
Overview of the experiment design. (a) Schematic illustration of one participant wearing an exosuit on two force plates that measure the ground reaction force. Motion capture cameras were placed around a participant. (b) Schematic illustration of each task.

To test the feasibility of the interaction force sensing system, five healthy subjects participated in the experiment (Table 1), performing three different tasks, as shown in Figure 6. The inclusion criteria for participation were: (1) age between 20 and 80 years, and (2) the ability to independently perform sit-to-stand, stand-to-sit, and walking tasks without assistance. The exclusion criteria were: (1) having body dimensions or anthropometric characteristics incompatible with properly wearing the wearable suit, and (2) experiencing discomfort or pain when performing movements with the wearable suit. During the experiment, a motion capture system tracked the position of reflective markers placed on the participant’s lower limb using 11 infrared cameras (model MX and Vantage, Vicon), as shown in Figure 6(a). Marker placement was based on the Helen Hayes marker set (Supplementary Figure S4), and the trajectory data were processed using Nexus software (Vicon) (Figure 4) to calculate the joint angles using Visual3D software (C-Motion, Inc.).

**Table 1.** Participant information

Participant	Age[Years]	Gender	Height [M]	Mass[kg]	Leg length(L/R) [m]
P1	25	M	1.73	66.1	0.85/0.84
P2	25	M	1.64	60.0	0.80/0.81
P3	66	M	1.67	61.0	0.82/0.82
P4	64	M	1.80	92.1	0.93/0.93
P5	65	F	1.65	59.8	0.82/0.82

The experiment involved three movement tasks: stand-to-sit and sit-to-stand (STS), overground walking (OW), and stair climbing (SC). The sit-to-stand task was segmented from the initiation of standing to the full knee extension, and the stand-to-sit task was segmented from the initiation of sitting to hip contact with the chair. For the overground walking and stair-climbing tasks, trials were segmented from the heel strike of the right foot to the subsequent heel strike of the same foot. To ensure natural movement patterns, participants performed one additional step before and after each measurement trial. These tasks were performed under three conditions: without wearing the robot (baseline), with the robot but in transparency mode (TM) where minimal tension is applied to prevent cable slack, and with the robot in assistive mode (AM) with predefined torque to assist motion (Figure 6(b)). Each participant performed three tasks under baseline, TM, and AM conditions, dedicating approximately 10 min to complete five cycles of each task (STS, OW, SC) using the wearable robot augmented with the proposed sensor system. This research design and protocol were approved by the Institutional Review Board of Korea Advanced Institute of Science and Technology (KH2021–220, approved on December 23, 2021).

### 2.3. Data analysis

#### 2.3.1. Net force and moment calculation from 3-DOF interaction forces

**Figure 7.**
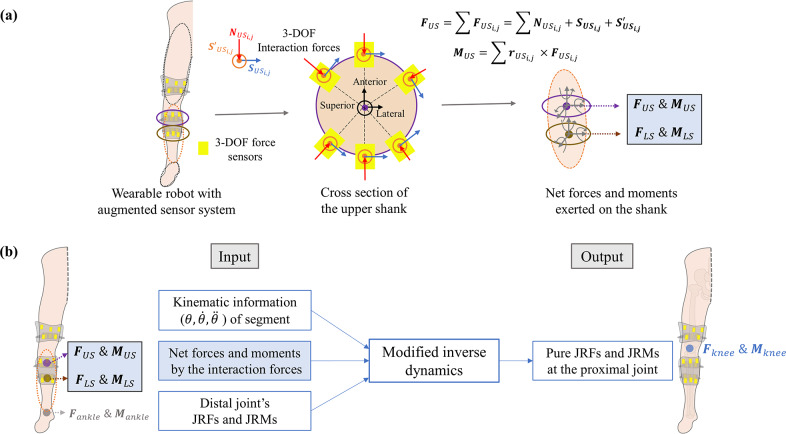
Conceptual figure for the modified inverse dynamics. (a) The procedure for calculating net forces and moments exerted on the shank from the measured 3-DOF interaction forces is shown, particularly highlighting the upper shank contact region with equations for deriving the net force **F_US_** and net moment **M_US_** exerted on the center point of the cross-section of the upper shank. (b) A schematic block diagram of the modified inverse dynamics. The distinct feature of the modified inverse dynamics, which considers interaction forces as input parameters in the calculation of JRF and JRM, is highlighted in the blue-colored block. An example illustration to obtain pure JRF **F_knee_** and JRM **M_knee_** at the knee joint by applying the modified inverse dynamics is shown.

To analyze the biomechanical effect of the wearable robot’s interaction forces on the wearer’s joints, the measured 3-DOF forces were converted into the net force and moment exerted on the right shank and thigh where the custom-built contact pads were located. The contact pads were divided into four regions based on superoinferior position: upper thigh, lower thigh, upper shank, and lower shank. The net forces and moments at the center of each contact region on the body segment were then derived from the resultant 3-DOF forces measured in that region. Figure 7(a) schematically illustrates the procedure for calculating the net forces and moments on the shank as an example. The 3-DOF forces of each sensor 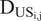 in the upper shank were denoted as 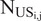 , 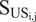 , and 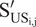 , representing normal, circumferential, and longitudinal directions to the upper shank, respectively. Using simplified body structure dimensions and predefined sensor positions within the contact pads, the net force 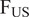 and the moment 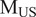 were formulated. The same process was applied to other contact regions. Detailed procedures are provided in Supplementary Note S1.

#### 2.3.2. Modified inverse dynamics

In this study, we propose “modified inverse dynamics” to extract pure JRFs and JRMs of human joints in the biomechanical analysis of the wearer’s movement assisted by the wearable robot. The conventional method (Chowdhury and Kumar, 2013; Caruntu and Moreno, 2019; Derrick et al., 2020; Baltzopoulos, 2024) employs body segment kinematics and external forces, such as ground reaction forces (GRF), in applying the recursive Newton-Euler algorithm (RNEA) to sequentially obtain JRFs and JRMs from the ankle to the hip joint.

In addition to utilizing the kinematic information and the distal JRFs and JRMs, the modified inverse dynamics approach incorporates interaction forces from the pHRI, as shown in Figure 7(b). Unlike the conventional method, which treats interaction forces as internal and unmeasured, the modified inverse dynamics approach explicitly incorporates these forces. This allows differentiation between the human body and the wearable robot, resulting in pure JRFs and JRMs at human joints. Figure 7(b) schematically illustrates this approach for the knee joint as an example. The net interaction forces and moments exerted on the shank ( 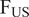 , 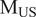 , 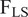 , and 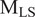 ), with “US” and “LS” denoting the upper and lower shank, respectively, were incorporated into the RNEA for the shank to derive the JRF and JRM at the knee. Detailed equations for the entire process, along with the comparisons between the modified and the conventional methods, highlighting the additional force/moment terms in the calculation of JRFs and JRMs, are provided in Supplementary Notes S2 and S3.

#### 2.3.3. Statistical analysis

The Wilcoxon signed-rank test and the Mann–Whitney U test were used to analyze the differences in the lower limb joint kinematics and the kinetics before and after applying the sensor data to the modified inverse dynamics approach using the average data across five task cycles. An analysis of JRFs and JRMs was conducted on the maximum and the minimum values in three directions.

## 3. Results

We calculated the JRFs and JRMs in three directions using the lower limb joint angles, the GRF, and the 3-DOF interaction force data. The forces and moments were normalized by body weight. The JRFs and JRMs at the hip and knee joints (Supplementary Figure S5) were compared during the three tasks (STS, OW, and SC) under five conditions: baseline, TM with the conventional inverse dynamics model, TM with the modified inverse dynamics model, AM with the conventional inverse dynamics model, and AM with the modified inverse dynamics model.

Significant differences in JRFs and JRMs were observed in the hip and knee joints during the three tasks when comparing the average maximum or minimum values calculated from the conventional and modified inverse dynamics under TM and AM conditions (Supplementary Figure S6). During the STS task, significant differences were observed in the maximum JRFs in lateral (AM: p = 0.032), medial (TM: p = 0.016, AM: p = 0.008), anterior (TM: p = 0.008, AM: p = 0.008), and posterior (TM: p = 0.034, AM: p = 0.016) directions and JRMs in extension (TM: p = 0.024, AM: p = 0.022), adduction (TM: p = 0.016, AM: p = 0.026) and abduction (TM: p = 0.034) directions in the hip joint. In the knee joint, significant differences were observed in the maximum JRFs in lateral (TM: p = 0.007, AM: p = 0.008) and medial (TM: p = 0.014, AM: p = 0.016) directions and JRMs in extension (AM: p = 0.049), adduction (AM: p = 0.034), and external rotation (AM: p = 0.044) directions. During the OW task, significant differences were found in the maximum JRFs in lateral (TM: p = 0.044, AM: p = 0.049) and medial (TM: p = 0.016, AM: p = 0.034) directions, and the maximum JRMs in internal (TM: p = 0.044) and external (TM: p = 0.016, AM: p = 0.042) rotation directions in the hip joint. Significant differences in the knee JRFs were observed in posterior (AM: p = 0.044), superior (TM: p = 0.046, AM: p = 0.049), and inferior (TM: p = 0.016, AM: p = 0.008) directions. In addition, significant differences in the knee JRMs were found in internal (TM: p = 0.010, AM: p = 0.008) and external (TM: p = 0.032, AM: p = 0.049) rotation directions. During the SC task, significant differences in the maximum JRM in internal rotation (AM: p = 0.042) direction at the hip joint and JRF in superior (TM: p = 0.042, AM: p = 0.049) and inferior (TM: p = 0.016, AM: p = 0.036) directions and JRM in flexion (TM: p = 0.036, AM: p = 0.026) directions at the knee joint were observed.

**Figure 8.**
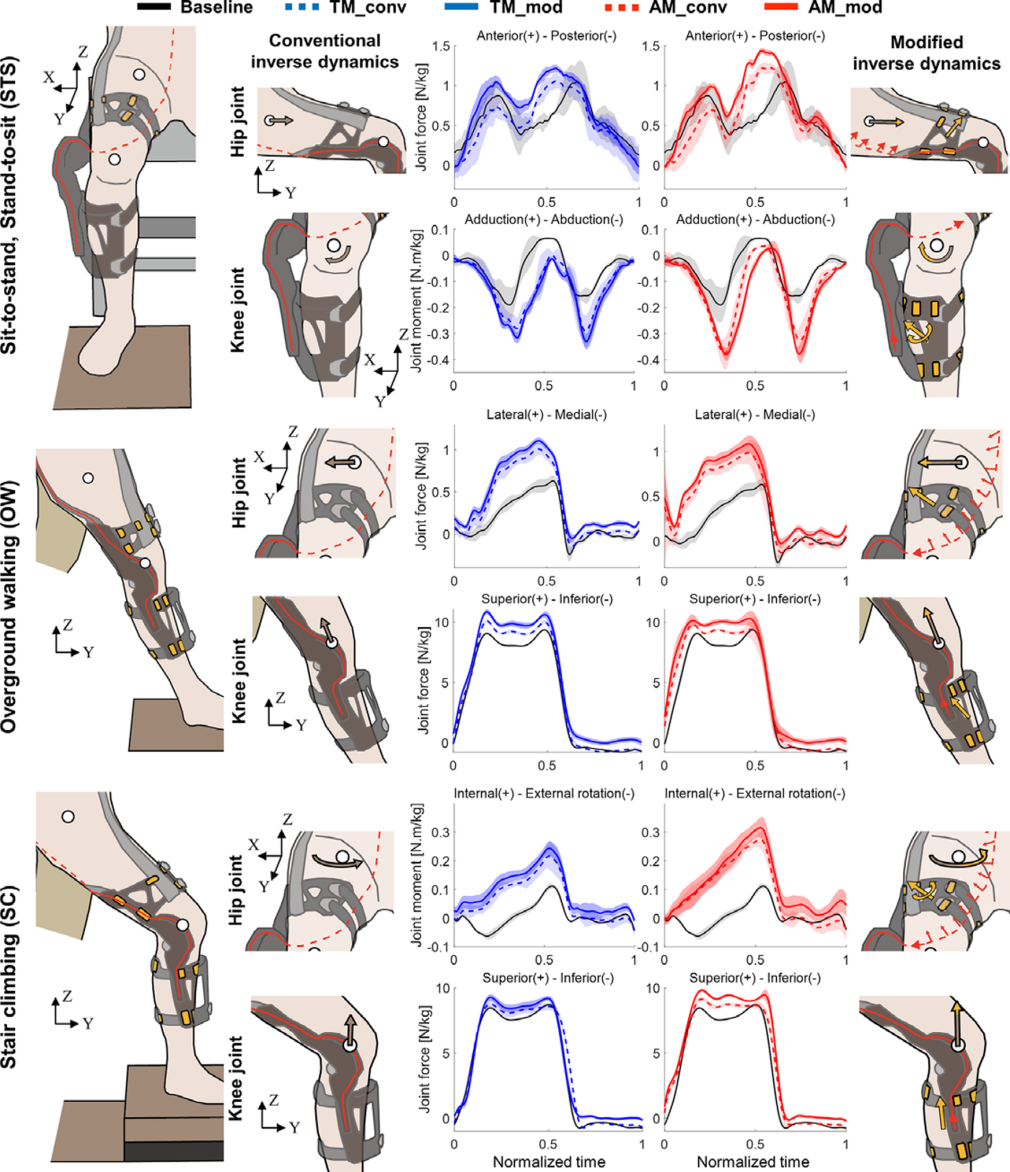
Joint force and moment trajectories observed significant differences in maximum/minimum values before and after applying sensor data during STS, OW, and SC tasks (p < 0.05). The shaded region represents ±1 standard deviation from each mean. TM and AM indicate transparency mode and assistive mode, respectively. Gray arrows indicate joint reaction forces and moments calculated from conventional inverse dynamics (conv) and yellow-extended arrows indicate joint reaction forces and moments calculated from modified inverse dynamics (mod) using sensor-measured forces and moments (yellow arrows). Joint reaction forces and moments were normalized to weight.

For the three tasks of STS, OW, and SC, representative cases were selected. These cases exhibited the most significant differences in the average maximum or minimum values of the three-directional JRFs and JRMs at the knee and hip joints. The corresponding trajectories are shown in Figure 8. To clearly illustrate the differences between the conventional and modified inverse dynamics approaches within each robot operation mode, the trajectories are presented in two separate graphs. The first graph shows the baseline conditions alongside the TM results, comparing the conventional (blue dashed line) and modified (blue solid line) inverse dynamics methods. Similarly, the second graph compares baseline conditions with the AM, showing both the conventional (red dashed line) and the modified (red solid line) inverse dynamics methods. The baseline trajectories are consistently represented by black solid lines. Incorporating the measured 3-DOF interaction forces in the modified inverse dynamics analysis resulted in higher magnitudes of JRFs and JRMs overall compared to the conventional inverse dynamics method.

## 4. Discussion

In this study, we developed a 3-DOF interaction force measurement system for estimating the pHRI effects on the joint forces and moments of the wearer. It extends the existing analyses from skin pressure or friction at the contact areas to joint-level biomechanics. Figure 8 shows significant differences in the JRFs and JRMs between the conventional (dotted lines) and modified (solid lines) inverse dynamics methods across TM (blue) and AM (red) conditions, with the no-exosuit case (black) as a baseline. Even in TM with minimal cable tension, wearing the wearable robot alters the peak JRFs and JRMs, which may be attributed to its added weight and structural constraints. In AM, where the cable tension actively assists movement, further changes in the JRFs and JRMs are observed. Importantly, the schematic illustrations, in Figure 8, emphasize how including (modified) or excluding (conventional) the interaction forces induced by cable routing and anchoring significantly affects the results. The conventional method underestimates the JRF/JRM values (gray arrows) in the directions where the interaction forces are applied. In contrast, our modified method accounts for these induced interaction forces (yellow arrows) from the cable tension (red arrows), enabling a more accurate estimation of the JRF and JRM values (yellow-extended arrows).

While the proposed sensor system demonstrated reliable performance during cyclic testing and human-subject trials, several practical considerations remain for long-term durability. The same sensor system was used across five participants, each performing approximately 2 hr of tasks, including donning and doffing of the sensor-augmented wearable robot (Figure 5(c)), totaling about 10 hr of continuous use under dynamic loading. No significant mechanical degradation was observed, although intermittent disconnections occasionally occurred at the soldered joints between the Hall effect sensors and wires due to repeated loading. These issues were readily resolved through re-soldering and did not impair overall functionality. The Hall effect sensor (WSH-135) also exhibits minor temperature-induced drift (0.3 mV/°C) and may respond to strong electromagnetic interference (EMI). During the experiment, temperature variation was naturally limited by continuous skin contact, and EMI was mitigated through the use of strong neodymium magnets and adequate distancing from electronic devices. Although the sensor system performed robustly under test conditions, further evaluations are necessary to ensure long-term durability and environmental robustness in real-world applications. If needed, the current sensor can be replaced with commercially available alternatives offering enhanced performance with reasonable cost considerations (Nasab et al., 2022).

We validated our measurement system by comparing the calculated interaction forces to the known cable tension of the Myosuit. The total interaction forces exerted on the lower limb, estimated by the vector summation, averaged approximately 149 N for STS and 135 N for gait tasks. Previous literature indicates cable tensions of approximately 160 N at level 2 assist during STS and 138 N at level 3 assist during gait (Haufe et al., 2020), the same assist levels used in our experiment. Since the cable tension is mostly transferred to the custom-built contact pads to assist both the knee and hip, our sensor system is suitable to measuring the interaction forces.

The feasibility of the system was demonstrated by understanding the observed differences in the JRFs and JRMs at the hip and knee, shown in Figure 8, attributed to the Myosuit’s cable-driven mechanism and the human movement dynamics. Originating from the medial side of the backpack module, the Myosuit cable wraps around the back of the thigh, passes through the lateral side, and anchors at the front-lateral side of the shank (Figure 1). The Myosuit actively assists both hip and knee extensions by tensioning this cable in a constant force during STS or modulated force patterns according to the knee angle during the early stance of walking.

Consequently, the anterior force at the hip joint during the STS movement increased along the JRF trajectory calculated using the modified inverse dynamics. In conventional inverse dynamics, a greater posterior force was computed at the hip joint than in the baseline condition, despite the exosuit applying an anterior force. However, in the modified inverse dynamics, incorporating interaction forces, the posterior force was not significantly different from the baseline condition when standing, while the anterior force increased during sitting and standing transitions. During OW and SC, where hip flexion was relatively small, the cable applied less anterior force, resulting in increased lateral JRF (OW) and internal rotation JRM with significant hip external rotation (SC). Although the exosuit assists hip extension in each movement, the increased hip joint forces and moments may potentially lead to an increased risk of joint-related injuries (Lenaerts et al., 2009; Harris et al., 2017), such as hip dislocation or osteoarthritis, if sustained over long-term use. This warrants further investigation using the modified inverse dynamics.

Additionally, the increased vertical loads on the knee joint during OW and SC can be attributed to the cable routing anchored at the front-lateral side of the shank. With a smaller range of knee flexion angle in walking compared to the STS, most of the cable tension was applied in the superoinferior direction to the knee joint. Therefore, the modified inverse dynamics analysis for the shank during OW and SC showed a larger vertical knee JRF compared to the conventional results and the baseline condition. During the STS task, where knee flexion was relatively large, larger lateral interaction forces were measured at the shank contact pad, resulting in an increased knee abduction JRM in the modified analysis. Such increased knee joint forces might increase the risk of arthritis over long-term use (Richards et al., 2018), highlighting the need for thorough validation and further studies before recommending prolonged use.

The forces and moments on the human joints due to the wearable robot can be calculated more accurately by using additional biomechanical features. Incorporating the interaction forces into the inverse dynamics proposed in this study is one of the methods for a precise analysis of joint forces and moments. Furthermore, measuring electromyography signals from the involved muscles may assist in estimating their contribution to compressive forces, ultimately enabling the prediction of joint contact or bone-on-bone forces (Baltzopoulos, 2024). This approach will more directly assess the risk of joint-related chronic conditions from long-term use of wearable robots (Messier et al., 2011). Additionally, the accuracy of individual biomechanical modeling can be enhanced through fluoroscopy techniques, such as magnetic resonance imaging (MRI), allowing for more precise dynamics analysis (Fernandez and Pandy, 2006).

## 5. Conclusion

This work proposes a method of accurately estimating JRFs and JRMs by incorporating 3-DOF interaction forces using a soft, compact, and lightweight sensor system embedded in contact pads. The original braces, pads, or cushions were replaced with custom-built contact pads closely modeled after their shapes and material properties. This allows the measurement system to be readily applied to various wearable robots beyond the Myosuit. The data acquisition module, mounted on the pad exterior, enables wireless transmission to avoid interference with the wearable robot and to support untethered movement. The modified inverse dynamics approach was proposed to take into account the measured interaction forces to obtain more accurate JRFs and JRMs. This enables precise biomechanical analysis and supports improved designs of wearable robots that can reduce the risk of joint-related chronic injuries.

## Supporting information

Cho et al. supplementary material Cho et al. supplementary material

## Data Availability

The data collected in the experiments are available from the corresponding authors upon reasonable request.

## Supplementary material

The supplementary material for this article can be found at http://doi.org/10.1017/wtc.2025.10014.
