# Supplementary material for: A soft 3-DOF interaction force measurement system for estimating the biomechanical effects of a soft wearable robot on the human joint
Source: Wearable Technol. 2025 Jul 15;6:e32. doi: 10.1017/wtc.2025.10014 (PMC12277210; doi:10.1017/wtc.2025.10014)
Supplement: Cho et al. supplementary material [file S2631717625100145sup001.docx]

**Supplementary Data**

**List of Contents**

**Supplementary Note S1.** Guidelines for net force and moment calculation

**Supplementary Note S2.** Procedure for the conventional inverse dynamics method

**Supplementary Note S3.** Procedure for the Modified Inverse Dynamics

**Supplementary Note S4.** System feasibility test about sensor system insertion

**Supplementary Table S1.** The root mean square difference (RMSD) of joint angles (%ROM) with and without sensor system insertion

**Supplementary Figure S1.** Cyclic test results of the Hall-effect based soft sensor

**Supplementary Figure S2.** Lower limb joint angle trajectories of P1 during three tasks with and without sensor system insertion

**Supplementary Figure S3.** Average lower limb joint angle trajectories during three tasks

**Supplementary Figure S4.** Motion capture markers and sensor modules placement

**Supplementary Figure S5.** Joint reaction force and moment trajectories during three tasks

**Supplementary Figure S6.** Average maximum/minimum joint forces and moments during three tasks

**Supplementary Figure S7.** Schematic procedure for obtaining net force and moment at the upper shank

**Supplementary Figure S8.** Application of the modified inverse dynamics to obtain accurate JRFs and JRMs of the human lower limb

**Supplementary Note S1. Guidelines for net force and moment calculation**

Consistent with the manuscript, the detailed procedures in this Supplementary material also illustrate the net force and moment applied to the center point of the upper shank as an example, as shown in Figure 6(a) and Figure S7.

Forces and moments in this section are expressed in the coordinate frame which parallels the segment coordinate system (SCS) frame of the shank and its origin coincides with $O_{\mathrm{US}}$, the center point of the upper shank’s cross-sectional circle. For convenience, we denote the lateral direction as $+x$ the anterior direction as $+y$, and the superior direction as $+z$. Each sensor surrounding the upper shank is numbered as $D_{\mathrm{US}_{i.j}}$, where US denotes its attachment location, the upper shank, the index i is 1 for the anterior and 2 for the posterior, and index j is 1 to 3 from the medial to lateral direction. For instance, the sensor positioned in the northeast direction in Figure S7 is noted $D_{\mathrm{US}_{1.3}}$. The force vector measured by the sensor $D_{\mathrm{US}_{i.j}}$ can be represented $\boldsymbol{F}_{US_{i,j}}= N_{US_{i,j}}\mathbb{i}_{i,j}+S_{US_{i,j}}\mathbb{j}_{i,j}+S_{US_{i,j}}^{'}\mathbb{k}_{i,j}$, where $N_{US_{i,j}}$, $S_{US_{i,j}}$, $S_{US_{i,j}}^{'}$ stand for the normal force (+ for the inward direction), circumferential shear force (+ for the lateral direction), and longitudinal shear force (+ for the superior direction), respectively. Also, the $\mathbb{i}_{i,j}, \mathbb{j}_{i,j}, \mathbb{k}_{i,j}$ are unit vectors of the sensor frame, each vector parallels the normal and bidirectional shear forces.

To sum up all force vectors from 3DOF sensors surrounding the upper shank, the measured forces with respect to each sensor frame have to be transformed into the shank SCS frame. The coordinate transformation from the $(\mathbb{i}_{i,j}, \mathbb{j}_{i,j}, \mathbb{k}_{i,j})$ to $(\mathbb{i, j, k)}$ in the SCS frame can be achieved by using a simplification of the human body structure and pre-defined sensor attachment positions. We assumed that the cross-sectional shape of the thigh and shank are circles, so the normal force vector of each sensor passes through $O_{\mathrm{US}}$. Also, we positioned each contact pad’s central sensor at the most anterior or posterior point of the segment and aligned the bidirectional shear forces with the lateral and superior directions of the SCS, when donning the wearable robot. Then, using the upper shank circumstance $C_{US}$ surrounded by the contact pad and widths $W_{US_{1}}, W_{US_{2}}$ of adjacent sensors attached to the front and back respectively, we can express the relationship between each sensor frame and the SCS by finding the central angles $\theta_{\mathrm{US}_{1}}=\frac{2\pi W_{US_{1}}}{C_{\mathrm{US}}} , \theta_{US_{2}}=\frac{2\pi W_{US_{2}}}{C_{\mathrm{US}}}$. The coordinate transformation for each sensor frame to SCS can be obtained by the following equations

$\mathbb{i}_{1,1}=\sin\theta_{\mathrm{US}_{1}}\mathbb{i-}\cos\theta_{\mathrm{US}_{1}}\mathbb{j}$, $\mathbb{j}_{1,1}=\cos\theta_{\mathrm{US}_{1}}\mathbb{i+}\sin\theta_{\mathrm{US}_{1}}\mathbb{j}$, $\mathbb{k}_{1,1}\mathbb{=k}$,

$\mathbb{i}_{1,2}\mathbb{=-j}$, $\mathbb{j}_{1,2}\mathbb{=i}$, $\mathbb{k}_{1,2}\mathbb{=k}$,

$\mathbb{i}_{1,3}=-\sin\theta_{\mathrm{US}_{1}}\mathbb{i-}\cos\theta_{\mathrm{US}_{1}}\mathbb{j}$, $\mathbb{j}_{1,3}=\cos\theta_{\mathrm{US}_{1}}\mathbb{i-}\sin\theta_{\mathrm{US}_{1}}\mathbb{j}$, $\mathbb{k}_{1,3}\mathbb{=k}$,

$\mathbb{i}_{2,1}=\sin\theta_{\mathrm{US}_{2}}\mathbb{i+}\cos\theta_{\mathrm{US}_{2}}\mathbb{j}$, $\mathbb{j}_{2,1}=\cos\theta_{\mathrm{US}_{2}}\mathbb{i-}\sin\theta_{\mathrm{US}_{2}}\mathbb{j}$, $\mathbb{k}_{2,1}\mathbb{=k}$,

$\mathbb{i}_{2,2}\mathbb{=j}$, $\mathbb{j}_{2,2}\mathbb{=i}$, $\mathbb{k}_{2,2}\mathbb{=k}$,

$\mathbb{i}_{2,3}=-\sin\theta_{\mathrm{US}_{2}}\mathbb{i+}\cos\theta_{\mathrm{US}_{2}}\mathbb{j}$, $\mathbb{j}_{2,3}=\cos\theta_{\mathrm{US}_{2}}\mathbb{i+}\sin\theta_{\mathrm{US}_{2}}\mathbb{j}$, $\mathbb{k}_{2,3}\mathbb{=k}$

The net force exerted on the upper shank, $\boldsymbol{F}_{US}$, is vector summation of 3DOF interaction forces measured by all sensors on the contact pad as follows:

$\boldsymbol{F}_{US}=F_{US_{x}}\mathbb{i+}F_{US_{y}}\mathbb{j+}F_{US_{z}}\mathbb{k} =\sum\boldsymbol{F}_{US_{i,j}}=\sum(N_{US_{i,j}}\mathbb{i}_{i,j}+S_{US_{i,j}}\mathbb{j}_{i,j}+S_{US_{i,j}}^{'}\mathbb{k}_{i,j})$, where

$\boldsymbol{F}_{US_{x}}=N_{US_{1,1}}\sin\theta_{\mathrm{US}_{1}}+S_{US_{1,1}}\cos\theta_{\mathrm{US}_{1}}+ S_{US_{1,2}}-N_{US_{1,3}}\sin\theta_{\mathrm{US}_{1}}+S_{US_{1,3}}\cos\theta_{\mathrm{US}_{1}}+N_{US_{2,1}}\sin\theta_{\mathrm{US}_{2}}+ S_{US_{2,1}}\cos\theta_{\mathrm{US}_{2}}+S_{US_{2,2}}-N_{US_{2,3}}\sin\theta_{\mathrm{US}_{2}}+S_{US_{2,3}}\cos\theta_{\mathrm{US}_{2}}$ ,

$\boldsymbol{F}_{US_{y}}=-N_{US_{1,1}}\cos\theta_{\mathrm{US}_{1}}+S_{US_{1,1}}\sin\theta_{\mathrm{US}_{1}}-N_{US_{1,2}}-N_{US_{1,3}}\cos\theta_{\mathrm{US}_{1}}-S_{US_{1,3}}\sin\theta_{\mathrm{US}_{1}}+N_{US_{2,1}}\cos\theta_{\mathrm{US}_{2}}-S_{US_{2,1}}\sin\theta_{\mathrm{US}_{2}}+N_{US_{2,2}}-N_{US_{2,3}}\cos\theta_{\mathrm{US}_{2}}+S_{US_{2,3}}\sin\theta_{\mathrm{US}_{2}}$ ,

$\boldsymbol{F}_{US_{z}}=S_{US_{1,1}}^{'}+S_{US_{1,2}}^{'}+S_{US_{1,3}}^{'}+S_{US_{2,1}}^{'}+S_{US_{2,2}}^{'}+S_{US_{2,3}}^{'}$,

The net moment exerted on the origin $O_{\mathrm{US}}$ of the upper shank, $\boldsymbol{M}_{US}$, can be calculated from cross-products of the position vector $\boldsymbol{r}_{US_{i,j}}$, from $O_{\mathrm{US}}$ to the center position of the sensor $D_{\mathrm{US}_{i.j}}$, and the force vector $\boldsymbol{F}_{US_{i,j}}$ as follows:

$\boldsymbol{M}_{US}=\sum\boldsymbol{M}_{US_{i,j}}=M_{US_{x}}\mathbb{i+}M_{US_{y}}\mathbb{j+}M_{US_{z}}\mathbb{k}=\sum\boldsymbol{r}_{US_{i,j}} \times\boldsymbol{F}_{US_{i,j}}=\sum(-R_{US}\mathbb{i}_{i,j}\times\left( N_{US_{i,j}}\mathbb{i}_{i,j}+S_{US_{i,j}}\mathbb{j}_{i,j}+S_{US_{i,j}}^{'}\mathbb{k}_{i,j} \right))=R_{US}\sum(-S_{US_{i,j}}^{'}\mathbb{j}_{i,j}+S_{US_{i,j}}\mathbb{k)}$ ,

where $R_{\mathrm{US}}=\frac{C_{US}}{2\pi}$ is the radius of the upper shank and each component can be computed as

$M_{US_{x}}=-R_{US} (S_{US_{1,1}}^{'}\cos\theta_{\mathrm{US}_{1}}+S_{US_{1,2}}^{'}+S_{US_{1,3}}^{'}\cos\theta_{\mathrm{US}_{1}}+S_{US_{2,1}}^{'}\cos\theta_{\mathrm{US}_{2}}+S_{US_{2,2}}^{'}+S_{US_{2,3}}^{'}\cos\theta_{\mathrm{US}_{2}})$,

$M_{US_{y}}=-R_{US} (S_{US_{1,1}}^{'}\sin\theta_{\mathrm{US}_{1}}-S_{US_{1,3}}^{'}\sin\theta_{\mathrm{US}_{1}}-S_{US_{2,1}}^{'}\sin\theta_{\mathrm{US}_{2}}+S_{US_{2,3}}^{'}\sin\theta_{\mathrm{US}_{2}})$,

$M_{US_{z}}=R_{US} (S_{US_{1,1}}+S_{US_{1,2}}+S_{US_{1,3}}+ S_{US_{2,1}}+S_{US_{2,2}}+S_{US_{2,3}})$.

The procedure of net force/moment calculation for other contact regions, Lower shank (LS), Upper thigh (UT), and Lower thigh (LT), is similar to the Upper shank (US) case explained above, except that no 3DOF sensors in the posterior side of the Upper thigh i.e.$\boldsymbol{F}_{UT_{2,j}}=0$, and no 3DOF sensor at the center of the frontal side of the Lower thigh i.e. $\boldsymbol{F}_{LT_{1,2}}=0$.

**Supplementary Note S2. Procedure for the conventional inverse dynamics method**

The conventional inverse dynamics refers to the recursive form of the Newton-Euler equations as follows:

$\boldsymbol{F}_{i}=m_{i}\boldsymbol{a}_{i}-m_{i}\boldsymbol{g}-\boldsymbol{F}_{i-1}$**,** $\boldsymbol{M}_{i}=\boldsymbol{I}_{i}\boldsymbol{\alpha}_{i}+\boldsymbol{\omega}_{i}\times\left( \boldsymbol{I}_{i}\boldsymbol{\omega}_{i} \right)-\boldsymbol{M}_{i-1}-\boldsymbol{r}_{C_{i},i}\times\boldsymbol{F}_{i}-\boldsymbol{r}_{C_{i-1}, i}\times\boldsymbol{F}_{i-1}$**,** i=1 to 3,

where descriptions of variables in the equations above are listed in the following table.

| Variable | Meaning |
| --- | --- |
| $\boldsymbol{F}_{\boldsymbol{i}}$ | joint reaction force (JRF) at the proximal joint i (c.f. $\boldsymbol{F}_{0}$ is ground reaction force) |
| $\boldsymbol{M}_{\boldsymbol{i}}$ | joint reaction moment (JRM) at the proximal joint i (c.f. $\boldsymbol{M}_{0}$ is ground reaction moment) |
| $\boldsymbol{m}_{\boldsymbol{i}}$ | mass of the body segment i |
| $\boldsymbol{a}_{\boldsymbol{i}}$ | (linear) acceleration of the center of mass |
| $\boldsymbol{g}$ | gravitational acceleration |
| $\boldsymbol{\omega}_{\boldsymbol{i}}$ | angular velocity of the segment |
| $\boldsymbol{\alpha}_{\boldsymbol{i}}$ | angular acceleration of the segment |
| $\boldsymbol{I}_{\boldsymbol{i}}$ | inertial tensor matrix of segment i with respect to the center of the mass |
| $\boldsymbol{C}_{\boldsymbol{i}}$ | center of the mass of segment i |
| $\boldsymbol{r}_{\boldsymbol{C}_{\boldsymbol{i}}\boldsymbol{,i}}$ | position vector from the center of mass of segment i to joint i |

As the GRF and GRM are known from the force plate, the recursive procedure of the Newton-Euler equation sets is applied in the order of the ankle, knee, and hip joint.

For JRF $\boldsymbol{F}_{ankle,conv}$ and JRM $\boldsymbol{M}_{ankle,conv}$ at the ankle:

$\boldsymbol{F}_{ankle,conv}=m_{foot}\boldsymbol{a}_{foot}-m_{foot}\boldsymbol{g}-\boldsymbol{F}_{GRF}$,

$\boldsymbol{M}_{ankle,conv}=\boldsymbol{I}_{foot}\boldsymbol{\alpha}_{foot}+\boldsymbol{\omega}_{foot}\times\left( \boldsymbol{I}_{foot}\boldsymbol{\omega}_{foot} \right)-\boldsymbol{M}_{GRF}-\boldsymbol{r}_{C_{foot}}\times\boldsymbol{F}_{GRF}-\boldsymbol{r}_{C_{foot}, ankle}\times\boldsymbol{F}_{ankle,conv}$, where ‘conv’ denotes the conventional inverse dynamics method.

For JRF $\boldsymbol{F}_{knee,conv}$and JRM $\boldsymbol{M}_{knee,conv}$ at the knee:

$\boldsymbol{F}_{knee,conv}=m_{shank}\boldsymbol{a}_{shank}-m_{shank}\boldsymbol{g}+\boldsymbol{R}_{foot,shank}\boldsymbol{F}_{ankle,conv}$,

$\boldsymbol{M}_{knee,conv}=\boldsymbol{I}_{shank}\boldsymbol{\alpha}_{shank}+\boldsymbol{\omega}_{shank}\times\left( \boldsymbol{I}_{shank}\boldsymbol{\omega}_{shank} \right)-\boldsymbol{R}_{foot,shank}\boldsymbol{M}_{ankle,conv}+\boldsymbol{r}_{C_{shank}, ankle}\times\boldsymbol{R}_{foot,shank}\boldsymbol{F}_{ankle, conv}-\boldsymbol{r}_{C_{shank}, knee}\times\boldsymbol{F}_{knee,conv}$, where $\boldsymbol{R}_{foot,shank}$ is the rotation matrix for the coordinate transformation from the foot SCS to the shank SCS.

For JRF $\boldsymbol{F}_{hip,conv}$ and JRM $\boldsymbol{M}_{hip,conv}$ at the hip:

$\boldsymbol{F}_{hip,conv}=m_{thigh}\boldsymbol{a}_{thigh}-m_{thigh}\boldsymbol{g}+\boldsymbol{R}_{shank, hip}\boldsymbol{F}_{knee,conv}$,

$\boldsymbol{M}_{hip,conv}=\boldsymbol{I}_{thigh}\boldsymbol{\alpha}_{thigh}+\boldsymbol{\omega}_{thigh}\times\left( \boldsymbol{I}_{thigh}\boldsymbol{\omega}_{thigh} \right)-\boldsymbol{R}_{shank, hip}\boldsymbol{M}_{knee,conv}+\boldsymbol{r}_{C_{thigh}, knee}\times\boldsymbol{R}_{foot,shank}\boldsymbol{F}_{knee, conv}-\boldsymbol{r}_{C_{thigh}, hip}\times\boldsymbol{F}_{hip,conv}$,

where $\boldsymbol{R}_{shank, hip}$ is the rotation matrix for the coordinate transformation from the shank SCS to the hip SCS.

**Supplementary Note S3. Procedure for the Modified Inverse Dynamics**

Compared to the conventional inverse dynamics, the modified inverse dynamics proposed in this paper includes the effect of 3DOF interaction forces to the Newton-Euler equations, as seen in Figure S8.

For JRF $\boldsymbol{F}_{ankle,mod}$ and JRM $\boldsymbol{M}_{ankle,mod}$ at the ankle, no interaction force terms are added since the wearable robot (Myosuit) does not contact with the human foot.

$\boldsymbol{F}_{ankle,mod}=m_{foot}\boldsymbol{a}_{foot}-m_{foot}\boldsymbol{g}-\boldsymbol{F}_{GRF}$,

$\boldsymbol{M}_{ankle,mod}=\boldsymbol{I}_{foot}\boldsymbol{\alpha}_{foot}+\boldsymbol{\omega}_{foot}\times\left( \boldsymbol{I}_{foot}\boldsymbol{\omega}_{foot} \right)-\boldsymbol{M}_{GRF}-\boldsymbol{r}_{C_{foot}}\times\boldsymbol{F}_{GRF}-\boldsymbol{r}_{C_{foot}, ankle}\times\boldsymbol{F}_{ankle,mod}$, where ‘mod’ denotes the modified inverse dynamics method.

For JRF $\boldsymbol{F}_{knee,mod}$ and JRM $\boldsymbol{M}_{knee,mod}$ at the knee, however, the net force/moment vectors $\boldsymbol{F}_{US}$, $\boldsymbol{M}_{US}$ exerted on the upper shank origin $O_{\mathrm{US}}$, and $\boldsymbol{F}_{LS}$, $\boldsymbol{M}_{LS}$ exerted on the lower shank origin $O_{\mathrm{LS}}$, as shown in Figure S8 should be included in the Newton-Euler equations as follows:

$\boldsymbol{F}_{knee,mod}=m_{shank}\boldsymbol{a}_{shank}-m_{shank}\boldsymbol{g}+\boldsymbol{R}_{foot,shank}\boldsymbol{F}_{ankle,mod}\boldsymbol{-}\boldsymbol{F}_{US}\boldsymbol{-}\boldsymbol{F}_{LS}$,

$\boldsymbol{M}_{knee,mod}=\boldsymbol{I}_{shank}\boldsymbol{\alpha}_{shank}+\boldsymbol{\omega}_{shank}\times\left( \boldsymbol{I}_{shank}\boldsymbol{\omega}_{shank} \right)-\boldsymbol{R}_{foot,shank}\boldsymbol{M}_{ankle,mod}+\boldsymbol{r}_{C_{shank}, ankle}\times\boldsymbol{R}_{foot,shank}\boldsymbol{F}_{ankle,mod}-\boldsymbol{r}_{C_{shank}, knee}\times\boldsymbol{F}_{knee,mod}\mathbf{-}\boldsymbol{M}_{US}\boldsymbol{-}\boldsymbol{M}_{LS}\boldsymbol{-}\boldsymbol{r}_{C_{shank}, US}\times\boldsymbol{F}_{US}\boldsymbol{-}\boldsymbol{r}_{C_{shank}, LS}\times\boldsymbol{F}_{LS}$,

where $\boldsymbol{r}_{C_{shank}, US}, \boldsymbol{r}_{C_{shank}, LS}$ means that the position vectors from the center of mass of the shank to $O_{\mathrm{US}}$ and $O_{\mathrm{LS}}$, respectively.

In the same way, to obtain JRF $\boldsymbol{F}_{hip,mod}$ and JRM $\boldsymbol{M}_{hip,mod}$ at the hip, $\boldsymbol{F}_{UT}$, $\boldsymbol{M}_{UT}$ and $\boldsymbol{F}_{LT}$, $\boldsymbol{M}_{LT}$ exerted on $O_{\mathrm{UT}}$ and $O_{LT}$ respectively are involved in the following equations:

$\boldsymbol{F}_{hip,mod}=m_{thigh}\boldsymbol{a}_{thigh}-m_{thigh}\boldsymbol{g}+\boldsymbol{R}_{shank, hip}\boldsymbol{F}_{knee,mod}\boldsymbol{-}\boldsymbol{F}_{UT}\boldsymbol{-}\boldsymbol{F}_{LT}$,

$\boldsymbol{M}_{hip,mod}=\boldsymbol{I}_{thigh}\boldsymbol{\alpha}_{thigh}+\boldsymbol{\omega}_{thigh}\times\left( \boldsymbol{I}_{thgih}\boldsymbol{\omega}_{thigh} \right)-\boldsymbol{R}_{shank, hip}\boldsymbol{M}_{knee,mod}+\boldsymbol{r}_{C_{thigh}, knee}\times\boldsymbol{R}_{shank, hip}\boldsymbol{F}_{knee,mod}-\boldsymbol{r}_{C_{thgih}, hip}\times\boldsymbol{F}_{hip,mod}\mathbf{-}\boldsymbol{M}_{UT}\boldsymbol{-}\boldsymbol{M}_{LT}\boldsymbol{-}\boldsymbol{r}_{C_{thigh}, UT}\times\boldsymbol{F}_{UT}\boldsymbol{-}\boldsymbol{r}_{C_{thigh}, LT}\times\boldsymbol{F}_{LT}$,

where $\boldsymbol{r}_{C_{thigh}, UT}, \boldsymbol{r}_{C_{thigh}, LT}$ means that the position vectors from the center of mass of the shank to $O_{\mathrm{UT}}$ and $O_{\mathrm{LT}}$, respectively.

To highlight additional terms in JRF and JRM calculation from the application of the modified inverse dynamics, the relationship between $\boldsymbol{F}_{mod}$ and $\boldsymbol{F}_{conv}$ as well as $\boldsymbol{M}_{mod}$ and $\boldsymbol{M}_{conv}$ are analyzed as follows:

JRF and JRM at the ankle are the same since no contact region in the foot.

$\boldsymbol{F}_{ankle,mod}=\boldsymbol{F}_{ankle,conv}$, $\boldsymbol{M}_{ankle,mod}=\boldsymbol{M}_{ankle,conv}$

JRF and JRM at the knee are altered as $\boldsymbol{F}_{US}, \boldsymbol{F}_{LS}, \boldsymbol{M}_{US}, \boldsymbol{M}_{LS}$ are added. By comparing each result of $\boldsymbol{F}_{knee,mod}, \boldsymbol{M}_{knee,mod}$ and $\boldsymbol{F}_{knee,conv}, \boldsymbol{M}_{knee,conv}$ from Note S2, we can get

$\boldsymbol{F}_{knee,mod}=\boldsymbol{F}_{knee,conv}\boldsymbol{-}\boldsymbol{F}_{US}\boldsymbol{-}\boldsymbol{F}_{LS}$,

$$\boldsymbol{M}_{knee,mod}=\boldsymbol{M}_{knee,conv}\mathbf{-}\boldsymbol{M}_{US}\boldsymbol{-}\boldsymbol{M}_{LS}\boldsymbol{-}\boldsymbol{r}_{knee,US}\times\boldsymbol{F}_{US}\boldsymbol{-}\boldsymbol{r}_{knee,LS}\times\boldsymbol{F}_{LS}$$

where $\boldsymbol{r}_{knee,US}, \boldsymbol{r}_{knee,LS}$ means that the position vectors from the knee joint to $O_{\mathrm{US}}$ and $O_{\mathrm{LS}}$, respectively.

Similarly, we can get the relationship between ($\boldsymbol{F}_{hip,mod}$ and $\boldsymbol{F}_{hip,conv}$) and ($\boldsymbol{M}_{hip,mod}$ and $\boldsymbol{M}_{hip,conv}$) by using $\boldsymbol{F}_{UT}, \boldsymbol{F}_{LT}, \boldsymbol{M}_{UT},\boldsymbol{M}_{LT}$ as well as $\boldsymbol{F}_{US}, \boldsymbol{F}_{LS}, \boldsymbol{M}_{US}, \boldsymbol{M}_{LS}$.

$\boldsymbol{F}_{hip,mod}=\boldsymbol{F}_{hip,conv}\boldsymbol{-}\boldsymbol{F}_{UT}\boldsymbol{-}\boldsymbol{F}_{LT}\boldsymbol{-}\boldsymbol{R}_{shank,thigh}\boldsymbol{F}_{US}\boldsymbol{-}\boldsymbol{R}_{shank,thigh}\boldsymbol{F}_{LS}$,

$\boldsymbol{M}_{hip,mod} =\boldsymbol{M}_{hip,conv}\mathbf{-}\left( \boldsymbol{M}_{UT}\boldsymbol{+}\boldsymbol{M}_{LT}\mathbf{+}{\boldsymbol{R}_{shank,thigh}\boldsymbol{M}}_{US}\boldsymbol{+}\boldsymbol{R}_{shank,thigh}\boldsymbol{M}_{LS} \right)-\boldsymbol{r}_{hip,UT}\times\boldsymbol{F}_{UT}+\boldsymbol{r}_{hip,LT}\times\boldsymbol{F}_{LT}-\boldsymbol{R}_{shank,thigh}\left( \boldsymbol{r}_{knee,US}\times\boldsymbol{F}_{US} \right)-\boldsymbol{R}_{shank,thigh}\left( \boldsymbol{r}_{knee,LS}\times\boldsymbol{F}_{LS} \right)-\boldsymbol{R}_{shank,thigh}\left( \boldsymbol{r}_{hip,knee}\times\boldsymbol{F}_{US} \right)-\boldsymbol{R}_{shank,thigh}(\boldsymbol{r}_{hip,knee}\times\boldsymbol{F}_{LS})$,

where $\boldsymbol{r}_{hip,UT}, \boldsymbol{r}_{hip,LT}$ denotes the position vectors from the hip joint to $O_{\mathrm{UT}}$ and $O_{\mathrm{LT}}$, respectively.

As shown in Figure S8, these differences occur because resultant forces/moments exerted on the segment alter the proximal joint’s reaction force and moment. Without involving $\boldsymbol{F}_{US}, \boldsymbol{F}_{LS}, \boldsymbol{M}_{US}, \boldsymbol{M}_{LS}$ in the equation of motion, the conventional inverse dynamics gives the combined segments’ JRF and JRM, not pure joint reaction forces or moments of the human shank. By considering interaction forces as external forces, the modified inverse dynamics enables us to split the human shank from the wearable robot’s shank part and get pure JRF and JRM at the knee joint. In the same way, the resultant force/moment terms $\boldsymbol{F}_{UT}, \boldsymbol{F}_{LT}, \boldsymbol{M}_{UT}, \boldsymbol{M}_{LT}$ have to be regarded as external forces and moments exerted on the thigh segment to obtain pure JRF and JRM of the hip joint of the human.

**Supplementary Note S4. System feasibility test about sensor system insertion**

We measured lower limb joint angles in three directions: extension/flexion, adduction/abduction, and internal/external rotation, during three tasks with and without the proposed 3-DOF force sensing system on the exosuit. The root mean square difference value normalized by the ROM of each joint angle between the two conditions, with and without the sensor system, was calculated to verify significant differences in the kinematic patterns due to the system attachment (Table S1).

After analyzing the three-direction angles in the three lower limb joints during the three tasks under two conditions, TM and AM, only 4 of the 54 joint angle data were significantly different: internal rotation (p = 0.038) during OW under AM condition, adduction (p = 0.047) during SC under TM condition in the knee joint, and adduction during OW under TM (p = 0.031) and AM (p = 0.008) conditions in the ankle joint. The speed of performing tasks like sitting and standing time in the STS and gait phase in the walking tasks were self-selected by the participant, with no significant differences observed with and without the sensor system (Fig. S2).

**Table S1. The root mean square difference (RMSD) of joint angles (%ROM) of P1 with and without sensor system insertion**

| Joint | | Sit-to-Stand (STS) | | | Overground walking (OW) | | | Stair climbing (SC) | | |
| --- | --- | --- | --- | --- | --- | --- | --- | --- | --- | --- |
|  |  | Exte-nsion | Add-uction | Internal rotation | Exte-nsion | Add-uction | Internal rotation | Exte-nsion | Add-uction | Internal rotation |
| Hip | TM | 2.50 (3.40) | 3.12 (3.67) | 2.48 (2.43) | 1.47 (1.44) | 1.94 (2.58) | 2.08 (2.56) | 1.03 (1.32) | 2.83 (2.66) | 1.95 (2.79) |
|  | AM | 3.54 (3.23) | 3.43 (3.85) | 4.22 (4.98) | 4.87 (5.05) | 3.61 (4.04) | 4.76 (5.37) | 1.70 (1.98) | 1.81 (2.09) | 2.15 (2.82) |
| Knee | TM | 3.87 (5.05) | 3.89 (5.17) | 6.30 (6.99) | 2.72 (3.70) | 2.70 (3.77) | 1.68 (2.21) | 1.50 (1.84) | **6.89 (7.47)** | 2.59 (2.49) |
|  | AM | 5.89 (5.97) | 4.25 (4.02) | 3.90 (4.46) | 6.06 (6.70) | 6.08 (6.67) | **6.83 (10.02)** | 2.57 (3.16) | 5.71 (5.81) | 2.02 (3.05) |
| Ankle | TM | 2.75 (3.57) | 4.72 (5.34) | 2.91 (3.08) | 0.83 (0.97) | **10.59 (8.61)** | 3.69 (4.11) | 1.16 (1.21) | 3.48 (3.83) | 3.03 (3.14) |
|  | AM | 3.59 (3.53) | 6.85 (6.65) | 3.11 (2.87) | 5.82 (5.71) | **9.49 (9.59)** | 6.63 (7.30) | 1.35 (1.31) | 2.91 (2.89) | 1.85 (2.03) |

Values are RMSD (standard deviation) of joint angles normalized by ROM. TM and AM indicate transparency mode and assistive mode, respectively. Significantly different data before and after inserting sensor system using Wilcoxon signed-rank test are indicated in bold (p<0.05).


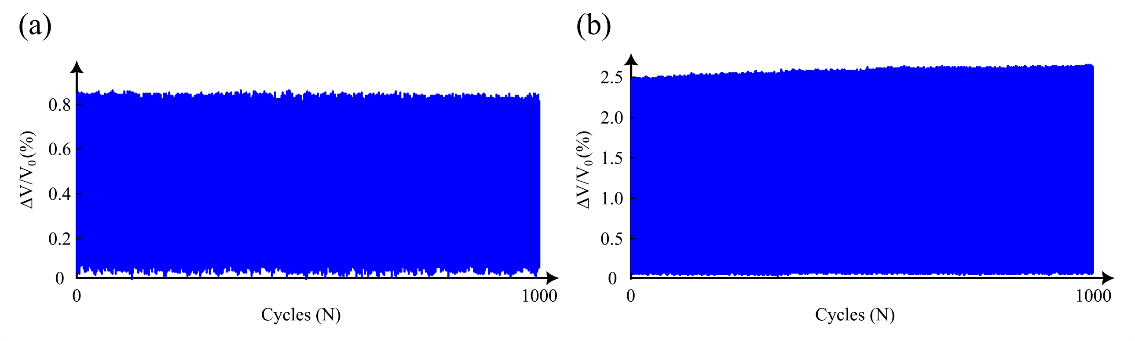

**Figure S1. Cyclic test results of the Hall-effect based soft sensor.** Each graph shows the (a) normal test and (b) shear test results for a single Hall-effect sensor embedded inside the sensor.

| 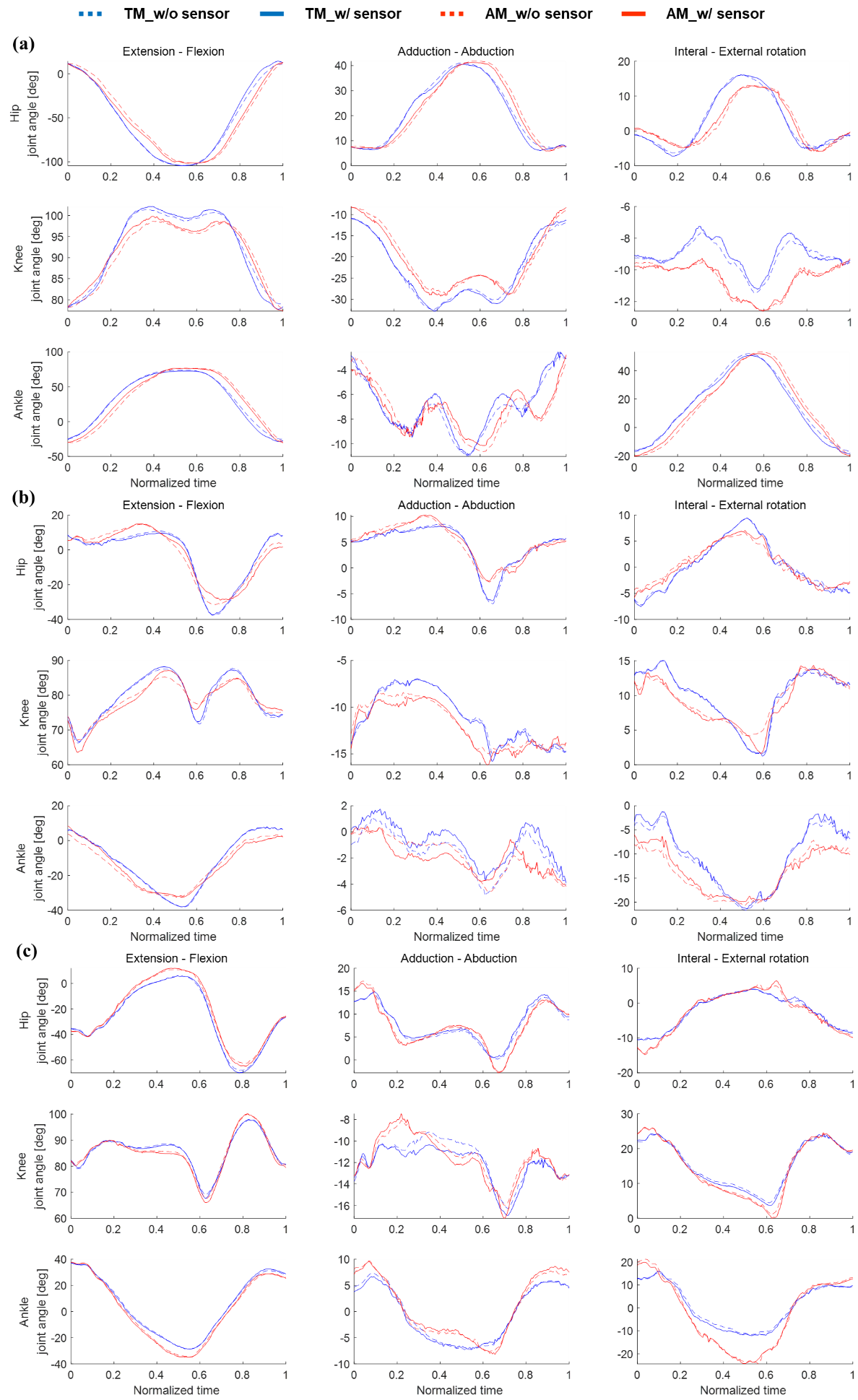  **Figure S2. Lower limb joint angle trajectories of P1 during three tasks with and without sensor system insertion.** (a) Sit-to-stand, (b) Overground walking, (c) Stair climbing. TM and AM indicate transparency mode and assistive mode, respectively. Dotted line and solid line indicate joint angle trajectory without and with sensor system insertion, respectively.  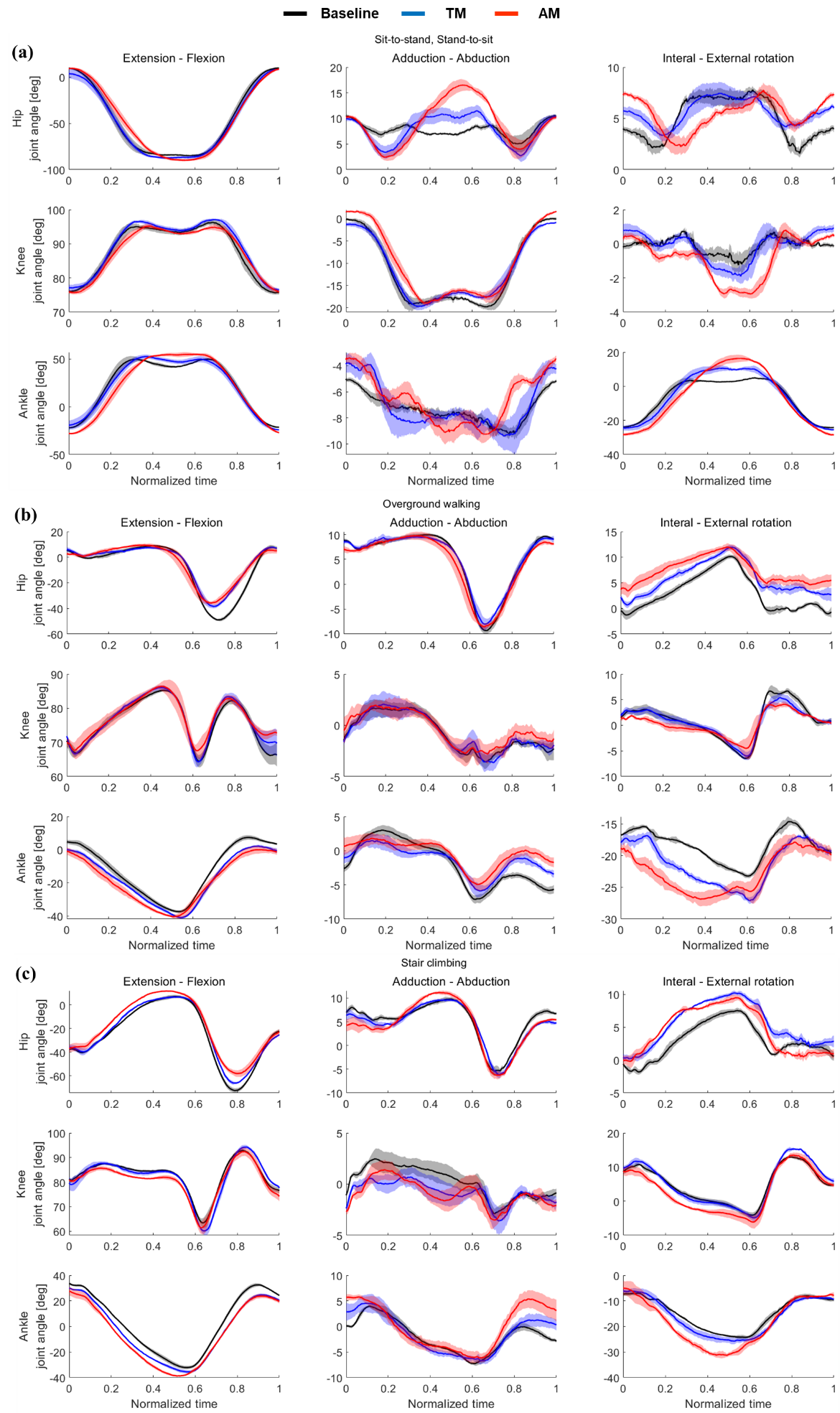  **Figure S3. Average lower limb joint angle trajectories during three tasks.** (a) Sit-to-stand, (b) Overground walking, (c) Stair climbing. TM (blue) and AM (red) indicate transparency mode and assistive mode, respectively. The shaded region represents ±1 standard deviation from each mean.  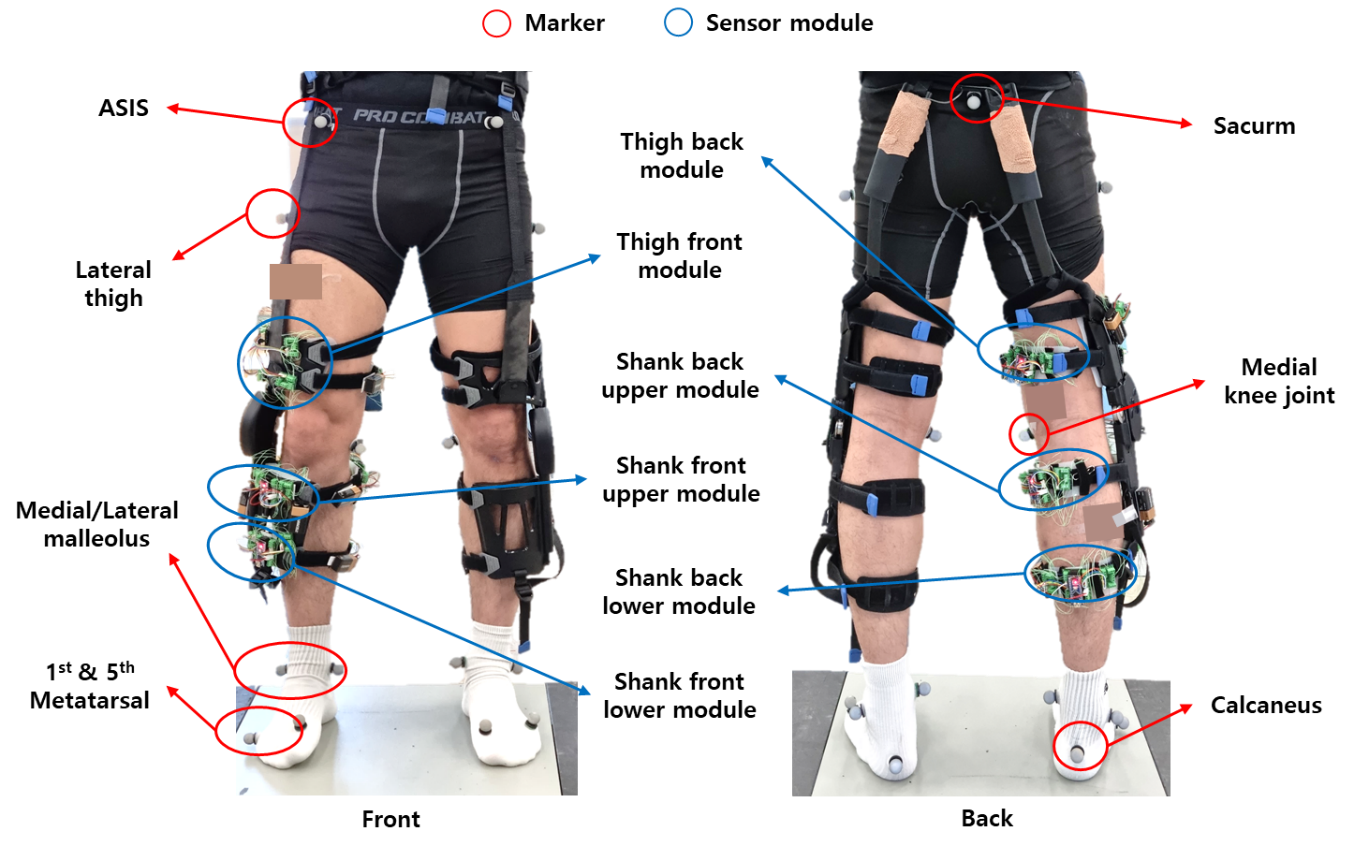  **Figure S4. Motion capture markers and sensor modules placement.** Three sensor modules and 17 reflective markers are attached on the lower limb or participants. The position of reflective markers followed the Helen Hayes marker set.  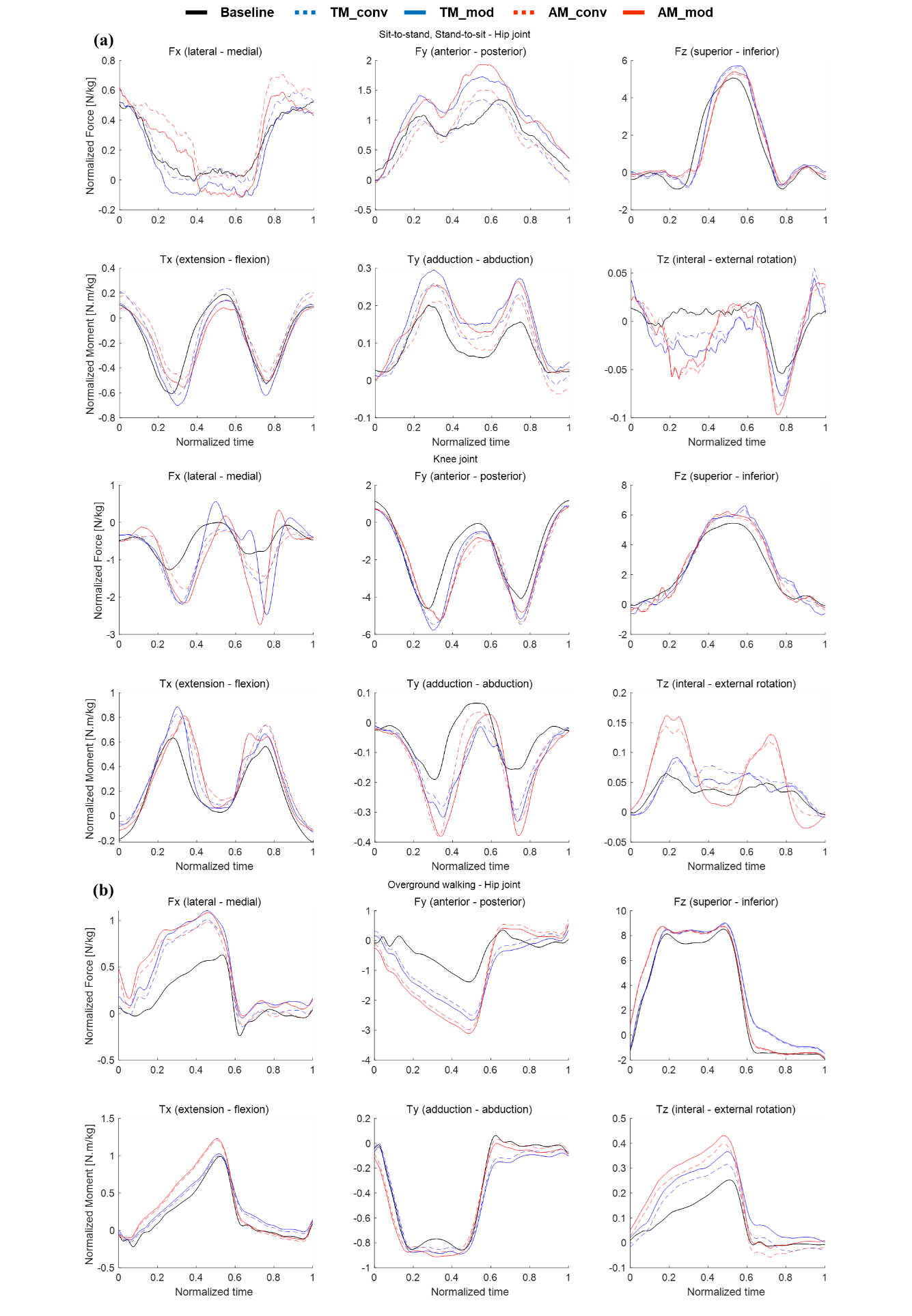  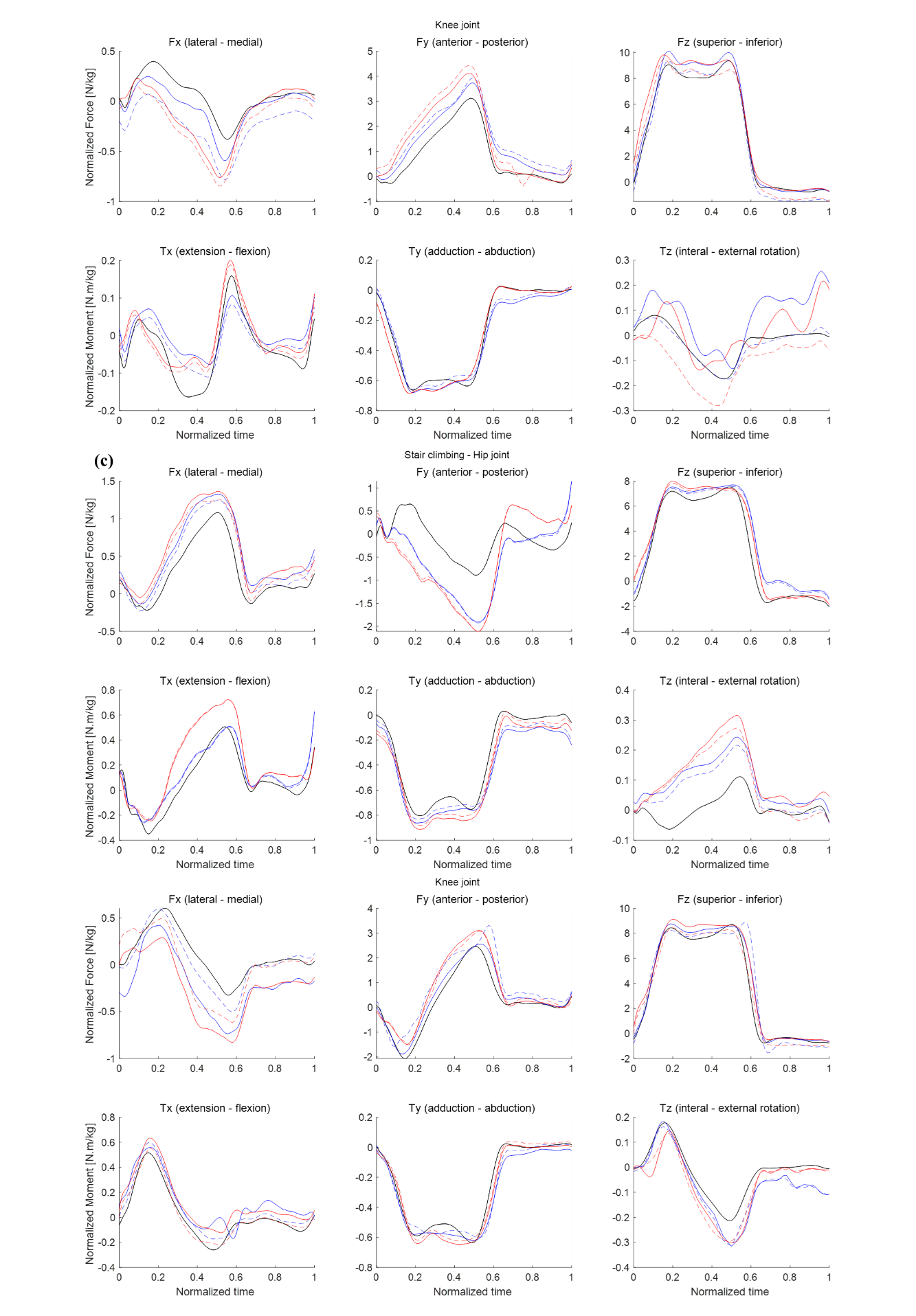  **Figure S5. Joint reaction force and moment trajectories during three tasks.** (a) Sit-to-stand, (b) Overground walking, (c) Stair climbing. TM and AM indicate transparency mode and assistive mod, respectively. Dotted line and solid line indicate joint reaction forces and moments using conventional and modified inverse dynamics, respectively.  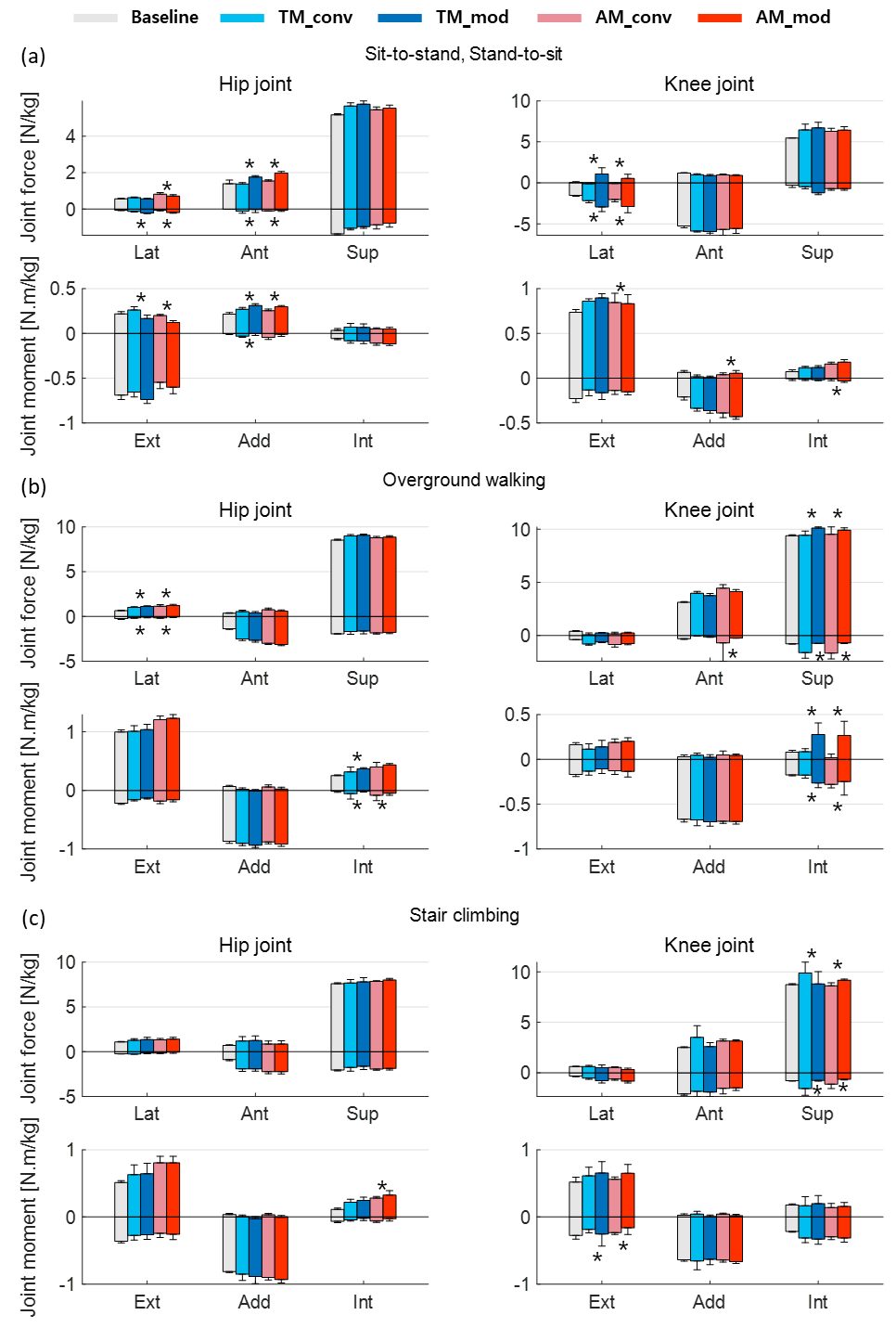  **Figure S6. Average maximum/minimum joint forces and moments during three tasks.** (a) Sit-to-stand, (b) Overground walking, (c) Stair climbing. Fx, Fy, and Fz indicate forces in the lateral(+)/medial(-), anterior(+)/posterior(-), and superior(+)/inferior(-) directions, respectively. Tx, Ty, and Tz indicate torques in the extension(+)/flexion(-), adduction(+)/abduction(-), and internal(+)/external(-) rotation directions, respectively. Forces and torques were normalized to weight. An asterisk (∗) indicates a significant difference before and after applying sensor data using Mann-Whitney U test (p < 0.05).  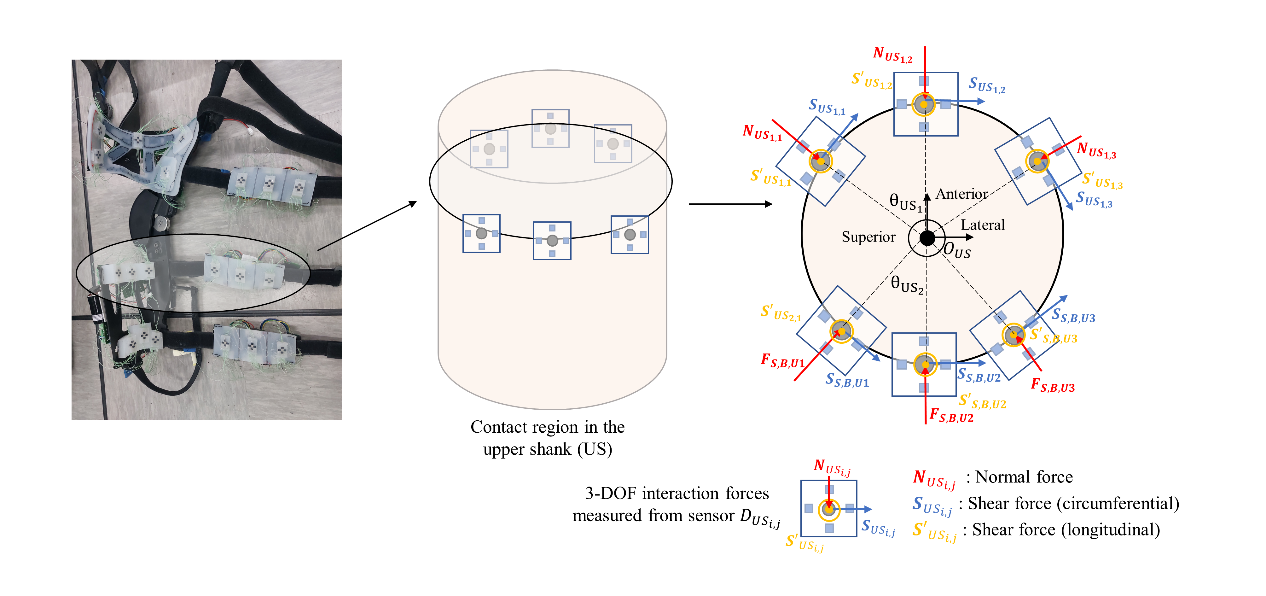 | |
| --- | --- |
| **Figure S7. Schematic procedure for obtaining net force and moment at the upper shank.** Six 3-DOF sensors are attached to the customized contact pads for the upper shank, with three sensors positioned on the anterior side and three on the posterior side. Each sensor frame is aligned parallel to the direction of the measured 3-DOF interaction forces, and the origin coincides with the center of the sensor position. The measured 3-DOF interaction forces from each sensor are transformed into the equivalent force and moment exerted on the origin $O_{\mathrm{US}}$, located at the center point of the upper shank cross-section enclosed by the contact pad. | |
| 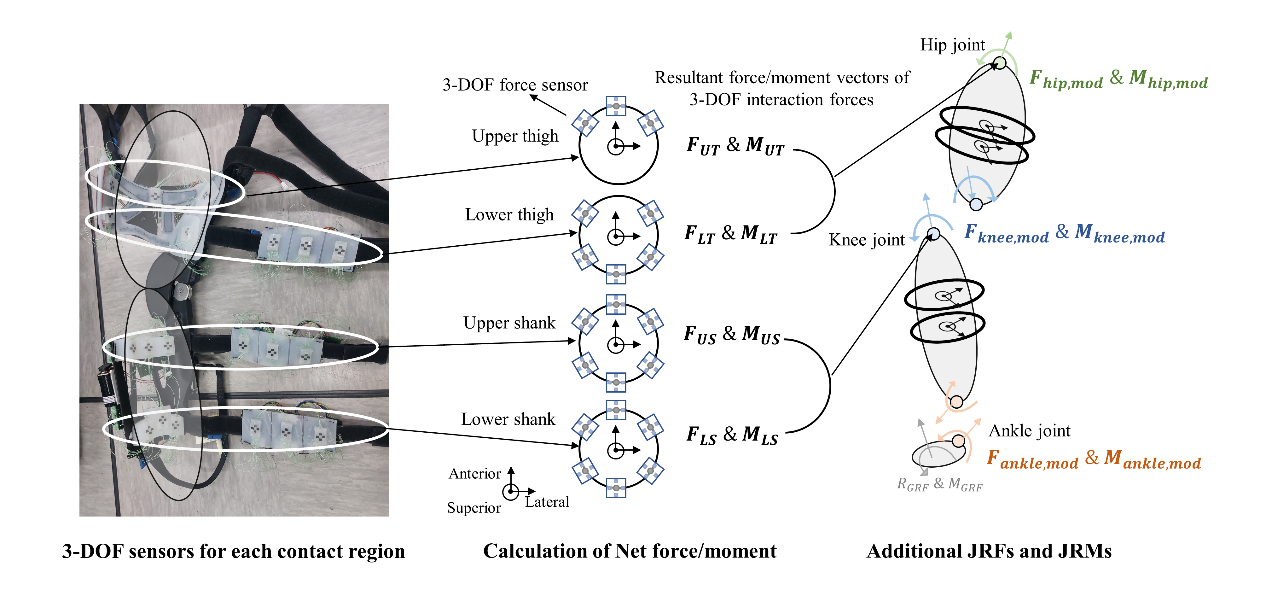 |  |
|  |  |
| **Figure S8. Application of the modified inverse dynamics to obtain accurate JRFs and JRMs of the human lower limb.** For the four contact regions—upper thigh, lower thigh, upper shank, and lower shank—resultant force and moment vectors exerted on each contact region are derived from the calculation procedure outlined in Supplementary Note S1. These force/moment terms are incorporated into the Newton-Euler equation for each body segment in the modified inverse dynamics. This method produces JRF and JRM at the proximal joint that includes additional effects from the interaction forces, leading to different results compared to the conventional method. |  |
